# Supplementary material for: Design and rationale of the Cardiovascular Health and Text Messaging (CHAT) Study and the CHAT-Diabetes Mellitus (CHAT-DM) Study: two randomised controlled trials of text messaging to improve secondary prevention for coronary heart disease and diabetes
Source: BMJ Open. 2017 Dec 21;7(12):e018302. doi: 10.1136/bmjopen-2017-018302 (PMC5778311; doi:10.1136/bmjopen-2017-018302)
Supplement: Supplementary file 1 [file bmjopen-2017-018302supp001.pdf]

Supplementary Materials

Design and rationale of the Cardiovascular Health and Text Messaging (CHAT) Study and CHAT-Diabetes Mellitus (CHAT-DM) Study: two randomized controlled trials of text messaging to improve secondary prevention for coronary heart disease and diabetes

TABLE OF CONTENTS

Page 2-3: CHAT and CHAT-DM studies site investigators by hospital

CHAT and CHAT-DM studies site investigators by hospital

1. China-Japan Union Hospital of Jilin University, Ping Yang, Zhaohui Feng, Mei Ding, Bing Li;
2. Dongfeng County Hospital, Wei Liu, Xiaoxin Li, Yanbin Zhang, Wen Cui, Sen Shi;
3. First Hospital of Shanxi Medical University, Qinghua Han, Liqin Duan, Chunrong Jin;
4. General Hospital of China FAW Group Corporation, Hongtao Pan, Lei Sun, Peng Chen;
5. Inner Mongolia Baogang Hopital, Yongdong Li, Liqing Jia, Xingxing Ren;
6. Jiangxi Provincial People's Hospital, Lang Hong, Ji Hong, Linfeng Li, Lihua Yuan, Yun Li;
7. Laixi People's Hospital, Xu Jiang, Hui Liu ,Guohao Xu, Yanrong Song;
8. Laoting County Hospital, Keyong Shang, Changjiang Liu, Kuituan Xi, Ying Yu;
9. Lujiang County People's Hospital, Kelian Xu, Cunqi Wang, Yongan Qian;
10. Nanyang Central Hospital, Shouzhong Yang, Yudong Li, Jianbu Gao, Songyu Zhang;
11. Port Hospital of Hebei Port Group Company, Ltd, Xia Wu, Penghui Yang, Xueqing Wang;
12. Qingdao Fuwai Hospital, Xianyan Jiang, Bin Zhang, Yumei Dong, Cheng Zheng, Wenchuan Hu;
13. Qinghai Cardiovascular and Cerebrovascular Hospital, Huiping Bian, Bo Chen, Xiaojuan Han, Na Han;
14. Qinzhou Second People's Hospital, Liyuan Chen, Qiuxia Liu, Lin Chen;
15. Quwo County People's Hospital, Xiwu Wang, Zhenlin Wu, Qiang Wang, Zhaohui Shangguan;
16. Shanxi Cardiovascular Hospital, Bin Yang, Yingting Yang;
17. Shenyang the Fourth Hospital of People, Yinjun Li, Wei Jiang;
18. Shuangcheng People's Hospital, Cheng Zhang, Aiguo Sun, Xuemei Wang;
19. Suzhou Kowloon Hospital, Shanghai Jiao Tong University School of Medicine, Feng Liu, Qiaoxia Zhou, Bingbing Zhu, Jie Guo;
20. The Affiliated Hospital of Qingdao University, Changyong Zhou, Yini Wang, Tao Yu, Zhe Su;
21. The First Affiliated Hospital of Fujian Medical University, Jinxiu Lin, Dajun Chai, Qinghui Guo;
22. The Affiliated Hospital of Xuzhou Medical University, Dongye Li, Yuanyuan Luo, Junhong Chen, Wei Qian, Qian Yu, Lulu Sun;
23. The First Hospital of Jilin University, Yang Zheng, Zhaoxi Liu, Lin Zou;
24. The Fourth Affiliated Hospital of China Medical University, Yuanzhe Jin, Xiaohong Zhang, Weina Hu;
25. The people's hospital of Dongxihu district of Wuhan ,Yongbo Lin, Ling Zhou, Hanliang Dan;
26. The People's Hospital of Liaoning Province, Zhanquan Li, Ying Liu, Dan Li;

27. The Second Affiliated Hospital of Xuzhou Medical College, Weiheng Wu, Li Li, Sai Zhang;
28. The Second Affiliated Hospital of Zhengzhou University, Xianen Fa, Lihua Zhang, Liqiang Sun, Youxu Jiang;
29. Tianjin Medical University General Hospital, Yuemin Sun, Bo Bian;
30. TEDA International Cardiovascular Hospital, Zhigang Liu, Zhipeng Guo, Cun Zhang;
31. Tongji Hospital of Tongji Medical College, Huazhong University of Science and Technology, Jiangang Jiang, Xiaoqing Shen, Ting Yu;
32. Wuhan Asia Heart Hospital, Xi Su, Songzhi Zhao, Wei Wu, Yujing Fan;
33. Wulate County People's Hospital, Jinlan Xu, Lei Xia, Yunmei Wang;
34. Xiangtan Central Hospital, He Huang, Jianping Zeng, Mingxing Wu, Yi Zhou;
35. Xinmin People's Hospital, Bo Jiang, Liwei Qi, Tongying Li;
36. Xuzhou First People's Hospital, Hongju Zhang, Chen Bian, Wei Li;
37. Zhengzhou Central Hospital, Lin Zhang, Yumei Guo;
