# Supplementary material for: Design and rationale of the Cardiovascular Health and Text Messaging (CHAT) Study and the CHAT-Diabetes Mellitus (CHAT-DM) Study: two randomised controlled trials of text messaging to improve secondary prevention for coronary heart disease and diabetes
Source: BMJ Open. 2017 Dec 21;7(12):e018302. doi: 10.1136/bmjopen-2017-018302 (PMC5778311; doi:10.1136/bmjopen-2017-018302)
Supplement: Author's manuscript [file bmjopen-2017-018302.draft_revisions.pdf]

BMJ Open

BMJ Open is committed to open peer review. As part of this commitment we make the peer review history of every article we publish publicly available.

When an article is published we post the peer reviewers' comments and the authors' responses online. We also post the versions of the paper that were used during peer review. These are the versions that the peer review comments apply to.

The versions of the paper that follow are the versions that were submitted during the peer review process. They are not the versions of record or the final published versions. They should not be cited or distributed as the published version of this manuscript.

BMJ Open is an open access journal and the full, final, typeset and author-corrected version of record of the manuscript is available on our site with no access controls, subscription charges or pay-per-view fees (http://bmjopen.bmj.com).

If you have any questions on BMJ Open's open peer review process please email info.bmjopen@bmj.com

Note from the Editor: Please note that there has been a minor change in the published article relating to the use of the Morisky scale to measure adherence. The authors explain this change in a linked response to the article (http://bmjopen.bmj.com/content/7/12/e018302.responses), which we have reproduced here:

Response to query regarding use of the Morisky scale

Xiqian Huo, Cardiologist, China Oxford Center for International Health Research, Fuwai Hospital, National Center for Cardiovascular Diseases

Other Contributors:

Erica S. Spatz

April 13, 2018

During the study design period, we planned to use a Chinese version of the Morisky scale to assess medication adherence. This version was previously validated and available for use in China. In 2017, we were informed by Dr.Morisky team that we need to pay to use the scale. After careful consideration, we opted not to use the Morisky scale and declined to pay the fee. These developments occurred during the submission and review process at BMJ Open. As such, the initial version of the manuscript noted the use of the Morisky scale to assess adherence, though this was deleted from the revised and published version. Instead, we will assess adherence using detailed medication information obtained during each follow-up visit.

BMJ Open

Design and rationale of the Cardiovascular Health and Text Messaging (CHAT) Study and CHAT-Diabetes Mellitus (CHAT-DM) Study: two randomized controlled trials of text messaging to improve secondary prevention for coronary heart disease and diabetes

Journal:	*BMJ Open*
Manuscript ID	bmjopen-2017-018302
Article Type:	Protocol
Date Submitted by the Author:	20-Jun-2017
Complete List of Authors:	Huo, Xiqian; Fuwai Hospital, National Center for Cardiovascular Diseases, State Key Laboratory of Cardiovascular Disease<br>Spatz, Erica; Yale University<br>Ding, Qinglan; Yale University<br>Horak, Paul ; Yale University<br>Zheng, Xin; Fuwai Hospital, National Center for Cardiovascular Diseases, State Key Laboratory of Cardiovascular Disease<br>Masters, Claire ; Yale University<br>Zhang, Haibo; Cardiovascular Institute & Fu Wai Hospital, Chinese Academy of Medical Sciences & Peking Union Medical College, China Oxford Centre for International Health Research<br>Irwin, Melinda; Yale School of Public Health<br>Yan, Xiaofang; Fuwai Hospital, National Center for Cardiovascular Diseases, State Key Laboratory of Cardiovascular Disease<br>Guan, Wenchi; Cardiovascular Institute & Fu Wai Hospital, Chinese Academy of Medical Sciences & Peking Union Medical College, China Oxford Centre for International Health Research,<br>Li, Jing; Cardiovascular Institute & Fu Wai Hospital, Chinese Academy of Medical Sciences & Peking Union Medical College, China Oxford Centre for International Health Research<br>Li, Xi; Fuwai Hospital, National Center for Cardiovascular Diseases, State Key Laboratory of Cardiovascular Disease<br>Spertus, John; Mid America Heart Institute of Saint Luke's Hospital<br>Masoudi, Frederick; Division of Cardiology, University of Colorado Anschutz Medical Campus<br>Krumholz, Harlan; Yale School of Public Health<br>Jiang, Lixin; Fuwai Hospital, National Center for Cardiovascular Diseases, State Key Laboratory of Cardiovascular Disease
**Primary Subject Heading**:	Research methods
Secondary Subject Heading:	Research methods, Medical management
Keywords:	Coronary heart disease < CARDIOLOGY, diabetes, text messaging, behavioral intervention, secondary prevention

SCHOLARONE™
Manuscripts

For peer review only

Design and rationale of the Cardiovascular Health and Text Messaging (CHAT) Study and CHAT-Diabetes Mellitus (CHAT-DM) Study: two randomized controlled trials of text messaging to improve secondary prevention for coronary heart disease and diabetes

Xiqian Huo, MD*; Erica S. Spatz, MD, MHS*; Qinglan Ding, MS; Paul Horak, BS; Xin Zheng, MD, PhD; Claire Masters, MHP; Haibo Zhang, MD; Melinda Irwin, PhD, MPH; Xiaofang Yan, MD; Wenchi Guan, MD; Jing Li, MD, PhD; Xi Li, MD, PhD; John A. Spertus, MD, MPH; Frederick A. Masoudi, MD, MSPH; Harlan M. Krumholz, MD, SM**; Lixin Jiang, MD, PhD**

(* Joint first authors)

(** Joint senior authors)

China Oxford Center for International Health Research, Fuwai Hospital, National Center for Cardiovascular Diseases, Chinese Academy of Medical Sciences and Peking Union Medical College, Beijing, People's Republic of China (X.H, X.Z, H.Z, X.Y, W.G, J.L, X.L, L.J);

Center for Outcomes Research and Evaluation, Yale University/Yale-New Haven Hospital, New Haven, Connecticut, USA (E.S.S, Q.D, P.H, C.M, M.I, H.M.K);

Health Outcomes Research, Saint Luke's Mid America Heart Institute/University of Missouri-Kansas City, Kansas City, Missouri, USA (J.A.S);

For peer review only

For peer review only - http://bmjopen.bmj.com/site/about/guidelines.xhtml

Division of Cardiology, University of Colorado Anschutz Medical Campus, Aurora, Colorado, USA (F.A.M).

Correspondence: Lixin Jiang, MD, PhD, National Clinical Research Center of Cardiovascular Diseases, Fuwai Hospital, National Center for Cardiovascular Diseases, 167 Beilishi Road, Xicheng District, Beijing, 100037, People's Republic of China. Tel: +86 10 8839 6203; Fax: +86 10 8836 5201. Email: jiangl@fwoxford.org

ABSTRACT

Introduction: Mobile health interventions have the potential to promote risk factor management and lifestyle modification, and are a particularly attractive approach for scaling across healthcare systems with limited resources. We are conducting two randomized trials to evaluate the efficacy of text-based health messages in improving secondary coronary heart disease (CHD) prevention among patients with or without diabetes.

Methods and analysis: The Cardiovascular Health And Text messaging (CHAT) and CHAT-Diabetes Mellitus (CHAT-DM) Studies are multi-center, single-blind, randomized controlled trials of text messaging versus standard treatment with 6 months of follow-up conducted in 37 hospitals throughout 17 provinces in China. The intervention group receives 6 text messages per week which target blood pressure control, medication adherence, physical activity, smoking cessation (when appropriate), glucose monitoring and lifestyle

For peer review only - http://bmjopen.bmj.com/site/about/guidelines.xhtml

recommendations including diet (in CHAT-DM). The text messages were developed based on behavioral change techniques, using models such as information-motivation-behavioral skills model, goal-setting and provision of social support. The estimated sample size is 790 in CHAT Study and 480 in CHAT-DM Study. In CHAT, the primary outcome is the change in systolic blood pressure (SBP) at 6 months. Secondary outcomes include a change in proportion of patients achieving a SBP<140mm Hg, low-density lipoprotein cholesterol (LDL-C), physical activity, medication adherence, body mass index (BMI), and smoking cessation. In CHAT-DM, the primary outcome is the change in glycemic hemoglobin (HbA_{1C}) at 6 months. Secondary outcomes include a change in proportion of patients achieving HbA_{1C}<7%, fasting blood glucose, SBP, LDL-C, BMI, physical activity and medication adherence.

Ethics and dissemination: The central ethics committee at the China National Center for Cardiovascular Disease (NCCD) approved the CHAT and CHAT-DM studies. Results will be disseminated via usual scientific forums including peer-reviewed publications.

Trial registration number: CHAT (NCT02888769) and CHAT-DM (NCT02883842); pre-results.

KEYWORDS: coronary heart disease, diabetes, text messaging, behavioral intervention, secondary prevention

For peer review only

For peer review only - http://bmjopen.bmj.com/site/about/guidelines.xhtml

Strengths and Limitations of study:

1. The main strengths of the study are that it evaluates the efficacy of an innovative, simple, scalable and cost-effective solution for improving secondary prevention of CHD. The trials are the first to investigate the effectiveness of text messages to support management of CHD and DM among a large and diverse population in China, and have the potential for scaling across healthcare systems in resource-constrained settings.
2. The study addressed the role of text messages in managing multiple risk factors in patients with CHD or DM. Moreover, there is currently little evidence about the effectiveness of such interventions for high-risk patients with coronary heart disease and diabetes.
3. The CHAT and CHAT-DM studies are further distinguished by their relative large sample sizes and culturally appropriate, theory-driven text messages. The messages were developed using behavior change technique and tailored to a specific Chinese patient population.
4. In contrast to most prior single center text-messaging trials, these two studies are conducted at 37 participating sites spread across a large and geographically diverse country, the results of which may be more generalizable.
5. However, medication adherence and physical activity are measured by self-report, which carries the possibility of recall bias and social desirability bias.

For peer review only - http://bmjopen.bmj.com/site/about/guidelines.xhtml

INTRODUCTION

The benefits of secondary prevention strategies for coronary heart disease (CHD) targeting lifestyle modification and risk factor management are well-established worldwide,[1, 2] however adoption of these strategies is suboptimal.[3] Smoking, inactivity and obesity are prevalent among people with established CHD and control of hypertension and diabetes are often suboptimal. Additionally, medication adherence is poor. Prior studies reveal that only one quarter of hospitalized patients do not fill all prescriptions within 120 days of hospital discharge.[4] Furthermore, less than half of patients hospitalized with acute myocardial infarction (AMI) are adherent to evidence-based medications 1 year later, with the greatest gaps in adherence occurring in the first 6 months after treatment initiation.[5-7]

In lower and middle-income countries (LMICs), including China, which face a growing burden of cardiovascular disease and greater challenges to medication access for secondary prevention, over two-thirds of patients with CHD take no medication.[8-10] While high medication costs are influential,[11] there is also limited time for education and consultation regarding lifestyle and medication management during clinic visits, which tend to be very brief.[12, 13] Therefore, innovative and cost-effective interventions to enhance adherence are urgently needed.

The pervasiveness of mobile phones to enhance the adoption of secondary prevention strategies for CHD in LMICs provides a promising opportunity. Mobile phones already serve as a primary, inexpensive and quick form of communication, alerts and reminders. In addition, the number of mobile phone

For peer review only - http://bmjopen.bmj.com/site/about/guidelines.xhtml

users has grown exponentially in the past decade worldwide, and is projected to reach 4.77 billion by 2017,[14] with nearly 200,000 messages sent every second.[15] As of August 2016, China had the largest number of mobile phone owners in the world, at 1.3 billion.[16] Mobile phones are also used across all geographic regions and income levels. Due to its ubiquity and convenience, mobile phone text messaging has the potential to be a scalable and powerful tool to deliver health information.[17, 18]

Prior studies of mobile phone text messaging have been conducted to improve glycemic control,[19] hypertension,[20] medication adherence,[21] as well as to promote smoking cessation,[22] and physical activity.[23] These trials have contributed important knowledge, and some, such as TEXT-ME[24] and TExT-MED[25] suggest that text-messaging interventions can influence patient behaviors and improve risk profiles. Still, several questions remain about the generalizability of these findings, especially for populations from LMICs. Most trials to date have been designed to target a single condition; yet patients with cardiovascular disease usually manage multiple conditions, requiring several lifestyle and treatment recommendations. Additionally, most studies of text messaging interventions have not been grounded in behavioral change techniques (BCT),[26, 27] which may influence the efficacy and generalizability of the intervention. Finally, most prior studies have been limited to single site interventions, many of which were underpowered and/or limited by selection bias. More data are needed to understand whether text-messaging interventions should be adopted as an effective strategy for supporting cardiovascular disease prevention among diverse populations from LMICs.

For peer review only - http://bmjopen.bmj.com/site/about/guidelines.xhtml

For peer review only

Accordingly, we designed and conducted the Cardiovascular Health And Texting (CHAT) and CHAT-Diabetes Mellitus (CHAT-DM) studies. The primary objective of these two studies is to evaluate the efficacy of an automated text message-based intervention, based on behavioral change techniques, in improving risk factor control and adoption of healthy lifestyle behaviors among patients with known CHD, with or without DM, who were discharged from multiple hospitals throughout China.

METHODS AND ANALYSIS

Study Overview

The CHAT and CHAT-DM studies are multi-center, single-blind, 2-arm, randomized controlled trials of an automated mobile phone text message-based intervention with 6 months of follow-up. Patients were recruited from 37 hospitals across 17 provinces in China (Figure 1). The enrollment of participants began on August 16, 2016. The two studies were registered at http://www.clinicaltrials.gov (NCT02888769 and NCT02883842) accordingly. All participants provided written informed consent at the initial trial visit.

Study Population

In CHAT, patients were eligible if they had established CHD defined as having a history of AMI and/or percutaneous coronary intervention (PCI), having access to a mobile phone to read and send text messages, and did not have

For peer review only - http://bmjopen.bmj.com/site/about/guidelines.xhtml

diabetes. In CHAT-DM, patients were eligible if they had a history of documented CHD (as defined in CHAT) and diabetes, and had access to a mobile phone to read and send text messages. In both studies, patients were excluded if they could not read or send text messages, had cognitive or communication disorders, or could not provide informed consent. A ‘screening log’ of basic demographic information and reasons for not participating in patients deemed ineligible but who declined to participate has been maintained.

Randomization and blinding

Participants were randomly allocated to either the intervention or control arms in a 1:1 ratio using a computerized randomization system. In order to achieve a balance of participants’ characteristics in both arms, we employed a stratified randomization approach, based on age, gender, AMI history, education degree and medical insurance type within each study. Researchers, statisticians and clinic staff were blinded to treatment allocation..

Trial intervention

Participants in the intervention groups of the CHAT and CHAT-DM studies receive semi-personalized text messages about CHD risk factor modification for 6 months as well as standard treatment (described below). The control group in both studies receive two thank-you text messages that contain no medical information each month as well as standard treatment. A training

For peer review only - http://bmjopen.bmj.com/site/about/guidelines.xhtml

For peer review only

session was held by research staff upon enrollment to ensure that all participants were capable of receiving, reading and sending text messages on their mobile phones. Participants were also instructed how to withdraw from the study should they desire, by responding with a specific character. Researchers could monitor the status of text message delivery, review responses sent by participants and manage withdrawal via a customized software platform. For instance, logs were kept to assess the proportion of text messages successfully delivered, the exact time of messages being sent and the reasons for those that failed to be sent.

CHAT and CHAT-DM intervention development

A bank of 550 text messages (280 in CHAT and 270 in CHAT-DM) was developed by a multidisciplinary team of cardiologists, endocrinologists, psychologists, nurses and public health researchers using a three-phase systematic and iterative approach (Figure 2). In CHAT study, text messages are categorized into five groups: (1) general education on CHD and AMI, (2) medication adherence, (3) blood pressure control, (4) physical activity, and (5) smoking cessation. The text messages in CHAT-DM study cover a range of diabetes self-management topics including: (1) general education on CHD and DM, (2) medication adherence, (3) glucose monitoring and control, (4) blood pressure control, (5) physical activity, and (6) lifestyle recommendations such as diet and foot care. All text messages are in Chinese, and each text message is less than 70 Chinese characters, which would be equivalent to about 140-160 character message in English.

For peer review only - http://bmjopen.bmj.com/site/about/guidelines.xhtml

Phase 1 Approach to developing text messages

Text messages were originally drafted by members of the research team in Chinese based on current guidelines[28-30] and standards of care pertaining to cardiovascular health and diabetes care. All messages were grounded in behavioral change techniques previously used to develop short health messages, providing information, goal-setting, motivation, social support and stress management advice.[31] Efforts were devoted to selecting the behavioral change techniques most applicable to the Chinese cultural context and compatible with Chinese beliefs and values (Table 1). For example, some text messages applied traditional Chinese aphorisms, chengyu, and catchy rhyme schemes in order to make them more acceptable to patients. As another example, in designing text messages that were motivating, especially in challenging situations, the multidisciplinary group felt that Chinese people tend to prefer more direct and structured counseling instructions rather than indirect and insight-oriented approaches (a category of psychotherapeutic approaches that promote individuals' adaptive behavior and course of action in life through understanding their internal motivation);[32] as such, text messages were written to provide practical approaches and real-life examples instead of abstract theories.[33, 34] Social and family-oriented goals were used more often than individual achievement to help improve health behaviors, consistent with cultural norms in China.[35, 36] These messages were sent to experts in behavioral change techniques and counseling for review and were used as examples to draft the initial bank of 550 text messages. Once drafted, all messages were reviewed, critiqued and revised within the internal research team.

For peer review only - http://bmjopen.bmj.com/site/about/guidelines.xhtml

Phase 2 Expert review

An expert panel made up of clinicians and academics reviewed each round of text message drafts with a different focus in each iteration. First, clinical experts (cardiologists, endocrinologists and psychologists) considered the accuracy, clarity and clinical benefit of each text message. Second, 21 messages from all categories in the CHAT and CHAT-DM studies text banks were randomly selected and translated into English by bilingual researchers. Experts in the fields of cardiology, endocrinology, epidemiology, psychology and behavioral science in the U.S. reviewed them and provided suggestions for further refinement. Finally, Chinese researchers and a linguist reviewed all 550 text messages, further refined the language, and paid special attention to the cultural meaning of all text messages to ensure better understanding among the targeted population (elderly Chinese patients with cardiovascular disease, with and without diabetes). Once feedback from the experts was addressed, the text bank was updated and prepared for user testing.

Phase 3 User testing and pilot study

For user testing, 19 randomly selected text messages from the finalized text bank in the CHAT and CHAT-DM studies were distributed to 39 individuals with CHD (with or without diabetes) for feedback. Likert-type items in a survey were used to assess usefulness and ease of understanding of the text messages. Open-ended questions were asked to obtain suggestions for improvement from participants. In total, 92.4% (582/630) rated the messages as easy to understand and 93.2% (587/630) rated the messages as useful. All 550 text messages were further modified based on feedback from user testing.

For peer review only - http://bmjopen.bmj.com/site/about/guidelines.xhtml

Results of the scores for the message categories in this phase are summarized in Table 2.

Next, the refined text message banks underwent pilot testing to evaluate the efficacy of the message delivery system and quality of the user experience. A customized software program sent messages through a gateway interface, allowing them to be sent to all individuals' mobile phones free of charge and at a bulk-rate cost (0.1 yuan per message, or .01 USD) to the research team. Participants from the user testing were enrolled in the pilot testing after providing written informed consent (n=33). For the pilot study, messages drawn from the entire text bank were sent to participants. At the end of the 1-month pilot study, 30 participants reported their experience of receiving messages and commented on frequency, timing, content and potential impact of text messages. Minor changes were made to the text message bank following the pilot study based on this feedback.

Frequency and timing of text message delivery

Each participant in the intervention group of the CHAT and CHAT-DM studies receives 6 text messages per week, randomly selected by the software system, across a 6 month timeframe. The messages are sent at one of three random times (9am, 12pm or 4pm), during weekends and weekdays (excluding Monday to give people a break). In the CHAT study, participants receive two general education messages on CHD (one if an active smoker), two blood pressure control messages, one medication adherence message, and one physical activity message per week. Smokers receive one smoking cessation message per week. Participants in the CHAT-DM intervention group receive

For peer review only - http://bmjopen.bmj.com/site/about/guidelines.xhtml

one general message, one blood pressure related message, one glucose control message, one lifestyle modification message, one medication adherence message and one physical activity message per week (Table 3).

Initially, a test message was sent to each participant to ensure that the correct mobile phone number had been recorded and the system was functioning appropriately. All participants received a personalized welcome message, a follow-up reminder and a birthday greeting while enrolled in the study. Most of the text messages were developed to be unidirectional and participants are not anticipated to reply, however bidirectional text messages, checking on medication adherence and blood pressure/glucose level measurements, are sent at weekly intervals to evaluate patient engagement. Throughout the 6-month follow-up, research staff call participants if they do not respond for two consecutive weeks, and only one call is made per patient during the intervention period, so as not to confound the intervention.

Procedures for Data Collection and Management

Data Collection

Basic and contact information (ID number, address, and phone number), anthropometric data (waist circumference, height and weight), resting blood pressure, heart rate, ambulatory blood pressure, and information on socioeconomic status, risk factor control and current medications were collected at baseline at hospital recruitment sites. Detailed information on patient outcomes (hospitalizations, discharge diagnoses, etc.) was also collected during the survey and hospital records or death certificates obtained

For peer review only - http://bmjopen.bmj.com/site/about/guidelines.xhtml

where necessary for adjudication. Additional assessments of baseline medication adherence (Morisky Medication Adherence Scales: MMAS-8),[37] physical activity (International Physical Activity Questionnaire: IPAQ),[38, 39] CVD-specific health status (Seattle Angina Questionnaire: SAQ),[40] health status (EuroQol five-dimensional questionnaire: EQ-5D),[41] and smoking status were also conducted in person. Lastly, blood and urine samples were collected for local lab tests and eventual transfer to the core lab in Beijing at the biobank of NCCD. Laboratory values including low-density lipoprotein cholesterol (LDL-C) and glycemic hemoglobin (HbA_{1C}) are assessed centrally. Follow-up information is conducted at 6 months by personal interviewing with research staff again collecting the information above (Table 4).

Data Management

A proprietary software platform was developed by the study IT team for use in sending text messages to participants. The platform is capable of sending tailored and semi-personalized text messages to participants and also recording responses. Additionally, this web-based platform can also serve to monitor project progress, as well as provide management support for hospitals, staff members, equipment and sampling materials. Trained medical staff members fill out pre-designed on-screen case report forms at each site, and data is then securely transmitted to the central server through automatic electronic transfer. To ensure the reliability and validity of the data, continuous checks are run to ensure that data being entered are complete and meet predefined data formats and ranges. The database is regularly backed-up and password protected so that only a limited number of approved staff members can access the data. In order to ensure the confidentiality of all personal

For peer review only - http://bmjopen.bmj.com/site/about/guidelines.xhtml

information, data confidentiality policies of the NCCD on data collection, storage and analysis have been strictly imposed.

Outcomes

In CHAT study, the primary outcome is the change in systolic blood pressure (SBP) after 6 months. Secondary outcomes include a change in proportion of patients achieving a SBP<140mm Hg, LDL-C, physical activity, medication adherence, body mass index (BMI), and smoking cessation. Exploratory outcomes include the prognosis of the patients at 6 months, such as death, non-fatal myocardial infarction, stroke and any re-hospitalization, as well as health status measured by SAQ and EQ-5D.

In CHAT-DM study, the primary outcome is the change in glycemic hemoglobin (HbA_{1C}) as measured by central blood sample. Secondary outcomes include a change in proportion of patients achieving HbA_{1C}<7%, fasting blood glucose, SBP, LDL-C, BMI, physical activity and medication adherence. Exploratory outcomes include prognosis of patients at 6 months, including death, nonfatal myocardial infarction, stroke and any re-hospitalization, as well as health status (SAQ and EQ-5D).

Blood pressure is measured on the right upper arm after 5 minutes of rest in a seated position using an electronic blood pressure monitor (Omron HEM-7111; Omron Corporation, Dalian, China). Two measurements are taken and the mean value is calculated. If the difference between the two SBP or diastolic blood pressure (DBP) readings is larger than 5 mm Hg, a third measurement is

For peer review only - http://bmjopen.bmj.com/site/about/guidelines.xhtml

done, and the mean value of the last two readings is calculated. HbA_{1C} is determined using a high-performance liquid chromatography technique with ADAMS^{TM}A_{1C}HA-8180 (ARKRAY, Inc, Japan). BMI is calculated by dividing weight in kilograms by height in meters squared. Physical activity is measured in metabolic equivalents of task (METs) per minute per week, using the short version of the IPAQ.[38, 39] Medication is assessed via MMAS-8, which scores the adherence from 0 to 8 points.[37] Smoking status is determined using either self-reported smoking status or urine-cotinine testing strip with a cut-off of 200ng/mL cotinine (COT Cotinine Test Colloidal Gold; Hangzhou Clongene Biotech Co., China).[42, 43] Quality of life is measured using Short Version of SAQ[40] and EQ-5D.[41]

Statistical Analysis

Intervention evaluation will be carried out on an intention-to-treat (ITT) basis. Values of analyzed endpoints between intervention group and control group will be compared using Student's t-tests for continuous variables or Chi-square tests for categorical variables according to the analysis plan. Mann-Whitney U tests will be used where continuous data are not normally distributed. The mean level of each risk factor will also be compared between groups in terms of relative risks, 95% CIs and two-sided p values for achieving the guideline level of each risk factor. We will follow prespecified analysis plan and subgroup analysis will be conducted accordingly.

All sample size calculations in both studies are for 80% power and 0.05 (one-sided) level of significance, allowing for 20% dropout rate during follow up.

For peer review only - http://bmjopen.bmj.com/site/about/guidelines.xhtml

In CHAT study, we estimate a mean SBP level of 132mm Hg [SD, 18mm Hg] in the control group according to the report of previous studies,[44] and assume an absolute reduction in SBP of 5 mm Hg at 6 months from baseline. Considering the compliance during the study intervention period, it was estimated that the mean SBP reduction in intervention group would be smaller. A total sample size of 790 patients was computed to detect a difference in SBP of 3.5mm Hg between two groups. In CHAT-DM study, we assume a mean HbA_{1C} level of 7.2% [SD, 1.6%] based on data from studies involving similar populations[45, 46] and supposed a 0.5% absolute decrease in HbA_{1C} across treatment group. A total sample size of 480 patients was estimated to detect a difference in HbA_{1C} of 0.4% between groups considering the potential compliance during the study. The trial results will be reported in accordance with the SPIRIT checklists.

ETHICS AND DISSEMINATION

The findings of the study will be disseminated by standard scientific forums, including peer-review publications and presentations at conferences. The central ethics committee at the China National Center for Cardiovascular Disease (NCCD) approved the CHAT and CHAT-DM studies. All collaborating hospitals accepted the central ethics approval except for 8, which obtained local approval by internal ethics committees. The Chinese government, which provides financial support, has no role in the design or conduct of the study, the collection, management, analysis, and interpretation of the data; or in the preparation or approval of articles resulting from the studies.

For peer review only - http://bmjopen.bmj.com/site/about/guidelines.xhtml

DISCUSSION

The CHAT and CHAT-DM studies aim to assess the efficacy of an innovative solution for improving secondary prevention of CHD by using simple and cost-effective text-messaging technology among patients with known CHD, with or without DM, throughout China. To the best of our knowledge, these two trials are the first to investigate the efficacy of text messages to support management of CHD and DM among a large population in China, and may set a model of such interventions that can be leveraged to improve risk factor management in resource-constrained settings.

The CHAT and CHAT-DM studies have several strengths. Few trials have addressed the role of text messages in managing multiple risk factors in patients with CHD or DM. Targeting multiple risk factors concurrently instead of managing a single risk factor may be more efficient and impactful on disease management as this strategy is more patient-centric than disease-centric, and have a greater likelihood of improving risk factor control and cardiovascular outcomes.[47, 48] Additionally, there is currently little evidence about the effectiveness of such interventions for high-risk patients with coronary heart disease and diabetes.

The CHAT and CHAT-DM studies are further distinguished by their large sample sizes and culturally appropriate, theory-driven text messages. Most text message intervention studies that have focused on health behavior or diabetes management have had sample sizes ranging from 18-357 participants.[49] CHAT has a sample size of around 790 while the sample size of

For peer review only - http://bmjopen.bmj.com/site/about/guidelines.xhtml

CHAT-DM is 480. It is noteworthy that CHAT is the first study using theory-based text messaging as a method of delivery for CHD patients' behavior change in China. Data from other text messaging intervention studies suggest that text messaging programs achieved better results when message content is theory-based,[49] however, few text messaging studies specified a theoretical rationale. Further, text messages were tailored to a specific Chinese patient population. Studies have found that the majority of Chinese adults prefer learning by following directive rules and guidelines,[50] and practical counseling instructions were provided through text messages to help them better modify health behaviors. Apart from this, the text message language was designed to be plain and easy to memorize, consistent with Chinese cultural features such as aphorism, chengyu and catchy rhyme schemes, making it more acceptable to patients.

The two studies have several additional strengths. In contrast to most prior single center text-messaging trials, these two studies are conducted at 37 participating sites spread across a large and geographically diverse country, the results of which may be more generalizable. Moreover, our research team devoted significant attention to quality data collection, including rigorous on-site monitoring of questionnaire answers, samples collection, and data management. Furthermore, an evaluation of the acceptability and feasibility of our text message-based intervention will provide further important evidence to inform future studies, particularly with regards to optimizing text content, text frequency, and the overall user experience.

Our study also has some potential limitations. First, medication adherence and physical activity are measured by self-report, which carries the possibility of

For peer review only - http://bmjopen.bmj.com/site/about/guidelines.xhtml

recall bias and social desirability bias. However, we believe that any such bias would be balanced across the treatment and control groups, and MMAS-8 and IPAQ have been validated and widely used in measuring this metric. Second, the text messages, though semi-personalized, were not tailored specifically to every individual, which may reduce their efficacy. These questions might be addressed in future studies using more individualized messages.

The CHAT and CHAT-DM studies have important public health implications. As low cost, non-pharmacological interventions, the CHAT and CHAT-DM studies may serve as important models for patient-centered, evidence-based public health interventions. LMICs, including China, face challenges with huge burdens of CVD and DM, limited health resources, and geographically and culturally diverse patient populations, making them the ideal places to conduct such studies. Still, high levels of mobile phone ownership across countries and income levels signal a promising new avenue for clinical research and that it may be possible to scale effective mobile health interventions for delivery to large populations in the coming years. If this innovative and simple prevention were proven to help, considering the low marginal cost and anticipated minimal adverse events, even with very modest effects, its benefits would be substantial in a country as large and populous as China.

In conclusion, the CHAT and CHAT-DM studies are multi-center, randomized controlled trials that are being conducted at 37 hospitals in China, and will thoroughly test the efficacy of text-messages to support secondary prevention for CHD and DM in LMICs. The studies targeted high-risk patient populations with a culturally-sensitive, scalable and cost-effective text-message intervention, went through comprehensive data collection and rigorous data

For peer review only - http://bmjopen.bmj.com/site/about/guidelines.xhtml

management, and have the potential to provide novel insights into disease management and be scaled-up to improve health in over a thousand patients in future.

Acknowledgments: We appreciate the multiple contributions made by study teams at the China Oxford Centre for International Health Research and the Yale-New Haven Hospital Center for Outcomes Research and Evaluation in the areas of study design and operations, particularly the contributions to the text message bank by Xuekun Wu, Si Xuan and Xiuling Wang, and data collection and analysis by Ying Sun, Chaoqun Wu, Xueke Bai, Jiamin Liu and Wuhanbilige Hundei. We appreciate the valuable advice from Clara Chow and her team in Sydney, Weigang Zhao, Geng Liu, Yuanlin Guo, Zhuo Xu and Zengwu Wang. We are also grateful for the support provided by the Chinese government.

Contributions: X.H, Q.D, P.H, X.Z, M.I, W.G, J.L, X.L, L.J: study concept design; X.H, E.S.S, Q.D, P.H, C.M, X.Z, H.Z, X.Y, J.A.S, F.A.M, H.M.K: drafting the initial manuscript and critical revision of the paper. All authors read and approved the final manuscript.

Funding: This project was supported by the Research Special Fund for Public Welfare Industry of Health (201202025, 201502009) from the National Health and Family Planning Commission of China, the National Key Technology R&D Program (2013BAI09B01, 2015BAI12B01, 2015BAI12B02) from the Ministry of Science and Technology of China and the 111 Project (B16005). Dr Spatz is

For peer review only - http://bmjopen.bmj.com/site/about/guidelines.xhtml

For peer review only

supported by grant K12HS023000 from the Agency for Healthcare Research and Quality Patient-Centered Outcomes Research Institute (PCORI) Mentored Career Development Program. The sponsors had no role in the preparation or approval of the manuscript.

Competing interests: Dr. Krumholz is a recipient of research agreements from Medtronic and from Johnson & Johnson (Janssen), through Yale University, to develop methods of clinical trial data sharing. He is also the recipient of a grant from the Food and Drug Administration and Medtronic to develop methods for post-market surveillance of medical devices; and is the founder of Hugo, a personal health information platform. The other authors have no potential conflicts to disclose.

Ethical approval: The central ethics committee at the China National Center for Cardiovascular Disease (NCCD) approved the CHAT and CHAT-DM studies. All collaborating hospitals accepted the central ethics approval except for 8, which obtained local approval by internal ethics committees.

REFERENCES

1. Gaede PH, Jepsen PV, Larsen JN, etc. The Steno-2 study. Intensive multifactorial intervention reduces the occurrence of cardiovascular disease in patients with type 2 diabetes. *Ugeskr Laeger*. 2003;165:2658-61.

2. Clark AM, Hartling L, Vandermeer B, etc. Secondary prevention programmes for coronary heart disease: a meta-regression showing the merits of shorter, generalist, primary care-based interventions. *Eur J Cardiovasc Prev Rehabil*. 2007;14:538-46.

For peer review only - http://bmjopen.bmj.com/site/about/guidelines.xhtml

3. Suaya JA, Shepard DS, Normand SL, etc. Use of cardiac rehabilitation by Medicare beneficiaries after myocardial infarction or coronary bypass surgery. *Circulation*. 2007;116:1653-62.
4. Jackevicius CA, Li P and Tu JV. Prevalence, Predictors, and Outcomes of Primary Nonadherence After Acute Myocardial Infarction. *Circulation*. 2008;117:1028-1036.
5. Akincigil A, Bowblis JR, Levin C, etc. Long-term adherence to evidence based secondary prevention therapies after acute myocardial infarction. *J Gen Intern Med*. 2008;23:115-21.
6. Jackevicius CA, Mamdani M and Tu JV. Adherence with statin therapy in elderly patients with and without acute coronary syndromes. *JAMA*. 2002;288:462-7.
7. Desai NR and Choudhry NK. Impediments to adherence to post myocardial infarction medications. *Curr Cardiol Rep*. 2013;15:322.
8. Yusuf S, Islam S, Chow CK, etc. Use of secondary prevention drugs for cardiovascular disease in the community in high-income, middle-income, and low-income countries (the PURE Study): a prospective epidemiological survey. *Lancet*. 2011;378:1231-43.
9. Krueger KP, Berger BA and Felkey B. Medication adherence and persistence: a comprehensive review. *Adv Ther*. 2005;22:313-56.
10. Urquhart J. The odds of the three nons when an aptly prescribed medicine isn't working: non-compliance, non-absorption, non-response. *Br J Clin Pharmacol*. 2002;54:212-20.
11. Walsh JM, Sundaram V, McDonald K, etc. Implementing effective hypertension quality improvement strategies: barriers and potential solutions. *J Clin Hypertens (Greenwich)*. 2008;10:311-6.
12. Peyrot M, Rubin RR, Lauritzen T, etc. Psychosocial problems and barriers to improved diabetes management: results of the Cross-National Diabetes Attitudes, Wishes and Needs (DAWN) Study. *Diabet Med*. 2005;22:1379-85.
13. Renders CM, Valk GD, Griffin S, etc. Interventions to improve the management of diabetes mellitus in primary care, outpatient and community settings. *Cochrane Database Syst Rev*. 2001:CD001481.
14. Portal TS. https://www.statista.com/statistics/274774/forecast-of-mobile-phone-users-wor

For peer review only - http://bmjopen.bmj.com/site/about/guidelines.xhtml

For peer review only

ldwide/ (accessed 20 Octorber 2016).
15. Union. IT. The World in 2010: ICT Facts and Figures. https://www.itu.int/ITU-D/ ict/material/FactsFigures2010.pdf 2010. 2010 (accessed 16 November 2016).
16. Ministry of Industry and Information Techonology of the People's Republic of China. Telecommunication industry situation of ecnomic operation in August 2016.https://www.miit.gov.cn/n1146312/n1146904/n1648372/c5295254/content.html. (accessed 10 November 2016).
17. RA A and I G-U. The role of mobile phones in increasing accessibility and efficiency in healthcare. *Vodafone Group Plc*. 2006:12-17.
18. Gurol-Urganci I, de Jongh T, Vodopivec-Jamsek V, etc. Mobile phone messaging reminders for attendance at healthcare appointments. *Cochrane Database Syst Rev*. 2013:CD007458.
19. Franklin VL, Waller A, Pagliari C, etc. A randomized controlled trial of Sweet Talk, a text-messaging system to support young people with diabetes. *Diabet Med*. 2006;23:1332-8.
20. Mills KT, Rubinstein A, Irazola V, etc. Comprehensive approach for hypertension control in low-income populations: rationale and study design for the hypertension control program in Argentina. *Am J Med Sci*. 2014;348:139-45.
21. Park LG, Howie-Esquivel J, Chung ML, etc. A text messaging intervention to promote medication adherence for patients with coronary heart disease: a randomized controlled trial. *Patient education and counseling*. 2014;94:261-8.
22. Free C, Knight R, Robertson S, etc. Smoking cessation support delivered via mobile phone text messaging (txt2stop): a single-blind, randomised trial. *Lancet*. 2011;378:49-55.
23. Maddison R, Whittaker R, Stewart R, etc. HEART: heart exercise and remote technologies: a randomized controlled trial study protocol. *BMC cardiovascular disorders*. 2011;11:26.
24. Chow CK, Redfern J, Hillis GS, etc. Effect of Lifestyle-Focused Text Messaging on Risk Factor Modification in Patients With Coronary Heart Disease: A Randomized Clinical Trial. *JAMA*. 2015;314:1255-63.
25. Arora S, Peters AL, Burner E, etc. Trial to examine text message-based mHealth in emergency department patients with diabetes (TExT-MED): a

For peer review only - http://bmjopen.bmj.com/site/about/guidelines.xhtml

randomized controlled trial. *Ann Emerg Med*. 2014;63:745-54 e6.
26. Patrick K, Raab F, Adams MA, etc. A text message-based intervention for weight loss: randomized controlled trial. *J Med Internet Res*. 2009;11:e1.
27. Hurling R, Catt M, Boni MD, etc. Using internet and mobile phone technology to deliver an automated physical activity program: randomized controlled trial. *J Med Internet Res*. 2007;9:e7.
28. [Chinese guidelines for prevention of cardiovascular diseases]. *Zhonghua Xin Xue Guan Bing Za Zhi*. 2011;39:3-22.
29. Wang JG. Chinese Hypertension Guidelines. *Pulse*. 2015;3:14-20.
30. Weng J, Ji L, Jia W, etc. Standards of care for type 2 diabetes in China. *Diabetes/Metabolism Research and Reviews*. 2016;32:442-458.
31. Abraham C and Michie S. A taxonomy of behavior change techniques used in interventions. *Health psychology : official journal of the Division of Health Psychology, American Psychological Association*. 2008;27:379-87.
32. Gelin M, Bugaiska A, Meot A , etc. Are animacy effects in episodic memory independent of encoding instructions? *Memory*. 2017;25:2-18.
33. Lin YN. The application of cognitive-behavioral therapy to counseling Chinese. *American journal of psychotherapy*. 2002;56:46-58.
34. Exum HA and Lau EYw. Counseling style preference of Chinese college students. *Journal of Multicultural Counseling and Development*. 1988;16:84-92.
35. Yeung GT and Fung HH. Social support and life satisfaction among Hong Kong Chinese older adults: family first? *European Journal of Ageing*. 2007;4:219-227.
36. Tseng W-S and Wu DY. *Chinese culture and mental health*: Academic Press; 2013.
37. Morisky DE, Green LW and Levine DM. Concurrent and predictive validity of a self-reported measure of medication adherence. *Med Care*. 1986;24:67-74.
38. Group I. International physical activity questionnaire. 2002.
39. NN Q and KJ L. Study on the reliability and validity of international physical activity questionnaire(Chinese vision,IPAQ). *Chin J Epidemiol*. 2004;25:265-268.
40. Chan PS, Jones PG, Arnold SA, etc. Development and validation of a

For peer review only - http://bmjopen.bmj.com/site/about/guidelines.xhtml

short version of the Seattle angina questionnaire. *Circ Cardiovasc Qual Outcomes*. 2014;7:640-7.

41. Wang H, Kindig DA and Mullahy J. Variation in Chinese population health related quality of life: results from a EuroQol study in Beijing, China. *Qual Life Res*. 2005;14:119-32.

42. Stevens KR and Munoz LR. Cigarette smoking: Evidence to guide measurement. *Res Nurs Health*. 2004;27:281-92.

43. Parker DR, Lasater TM, Windsor R, etc. The accuracy of self-reported smoking status assessed by cotinine test strips. *Nicotine Tob Res*. 2002;4:305-9.

44. Hu D, Li J, Li X, etc. Investigation of blood lipid levels and statin interventions in outpatients with coronary heart disease in China: the China Cholesterol Education Program (CCEP). *Circ J*. 2008;72:2040-5.

45. Bi Y, Zhu D, Cheng J, etc. The status of glycemic control: A cross-sectional study of outpatients with type 2 diabetes mellitus across primary, secondary, and tertiary hospitals in the Jiangsu province of China. *Clin Ther*. 2010;32:973-83.

46. Jinping Z, Lixia J, Wenhui Z, etc. Relationship between personal medical care and microvascular complications in patients with type 2 diabetes mellitus in Beijing. *Chin J Diabetes Mellitus*. 2016;8:463-467.

47. Smith SC, Jr. Current and future directions of cardiovascular risk prediction. *Am J Cardiol*. 2006;97:28A-32A.

48. Weingarten SR, Henning JM, Badamgarav E, etc. Interventions used in disease management programmes for patients with chronic illness-which ones work? Meta-analysis of published reports. *BMJ*. 2002;325:925.

49. Hall AK, Cole-Lewis H and Bernhardt JM. Mobile Text Messaging for Health: A Systematic Review of Reviews. *Annual review of public health*. 2015;36:393-415.

50. Littrell R. Teaching students from Confucian cultures. 2005.

For peer review only

For peer review only - http://bmjopen.bmj.com/site/about/guidelines.xhtml

Figure Legends

Figure 1. Geographic distribution of sites in CHAT and CHAT-DM studies

Figure 2. Text Development Process

For peer review only

For peer review only - http://bmjopen.bmj.com/site/about/guidelines.xhtml

Table 1. Behavior change techniques used in message development and example messages

Behavior change technique[31]	Content/explanation[31]	Example text message in Chinese and English
Provide information about behavior-health link	General information about behavioral risk	The way you cook can impact your health as well. Steaming, boiling and sautéing are better ways to cook than deep-frying and pan frying. Cooking in less oil is a healthy alternative.
Prompt barrier identification	Identify barriers to performing the behavior and plan ways of overcoming them	Taking diabetes medications and injecting insulin regularly can help control your blood sugar. Forgetting to take your medication? Try to set a repeating alarm on your cell phone to remind to you take your medication or insulin injection.
Set graded tasks	Set easy tasks, and increase difficulty until target behavior is reached.	Have you been finding it hard to quit smoking? In the beginning things are always hard. You can use a schedule to gradually reduce the number of cigarettes you smoke. For example, you

For peer review only - http://bmjopen.bmj.com/site/about/guidelines.xhtml

		may try going from 20 cigarettes to 15 per day for a week.
Provide instruction	Telling the person how to perform a behavior and/or preparatory behaviors	If you experience symptoms of angina (severe chest pain), place one nitroglycerin tablet under your tongue. Sit, stay calm and rest if you ever forget medications while going out. If angina symptoms are not relieved within 10 minutes, seek medical attention immediately.
Prompt self-monitoring of behavior	The person is asked to keep a record of specified behavior(s).	A cold or diarrhea will make your blood sugar levels rise, so monitor your glucose more frequently when you are sick. If you are using insulin, test your blood glucose 6-8 times a day, keep a blood glucose log and share it with your health providers.
Prompt practice	Prompt the person to rehearse and repeat the behavior or preparatory behaviors	As an old Chinese saying goes, 'It takes more than one cold day for a river to freeze three feet deep; ice in the river takes a long time to melt.' Similarly, cerebrovascular disease requires

For peer review only - http://bmjopen.bmj.com/site/about/guidelines.xhtml

		long-term prevention and treatment. Remember to take your medications as prescribed!
Plan social support or social change	Prompting consideration of how others could change their behavior to offer the person help or (instrumental) social support.	Quitting smoking on your own can be difficult. Tell your friends and family when you are quitting so that they will stop giving you cigarettes. Support and encouragement from your loved one can be helpful as well.
Stress management	May involve a variety of specific techniques (e.g., progressive relaxation) that do not target the behavior but seek to reduce anxiety and stress	Relaxation is something we need to learn and practice. Listening to music, reading, or talking to friends and family can ease stress.
Motivational interviewing	Prompting the person to provide self-motivating statements and	Did you smoke less today than you did yesterday or days before? If you did reduce the amount of cigarettes, it is something worth

For peer review only - http://bmjopen.bmj.com/site/about/guidelines.xhtml

evaluations of their own behavior to minimize resistance to change	celebrating. We are sure that you have put a lot of effort into quitting. Keep up the good work and you can make a difference!

For peer review only

For peer review only - http://bmjopen.bmj.com/site/about/guidelines.xhtml

Table 2. Survey scores for various message categories in user test

	Strongly agree or agree	Neutral	Strongly disagree or disagree
**General health messages**			
Information easy to understand (n=142)	130 (91.6%)	6 (4.2%)	6 (4.2%)
Information was useful (n=142)	129 (90.9%)	9 (6.3%)	4 (2.8%)
**Hypertension**			
Information easy to understand (n=181)	168 (92.8%)	7 (3.9%)	6 (3.3%)
Information was useful (n=181)	169 (93.4%)	6 (3.3%)	6 (3.3%)
**Medication Adherence**			
Information easy to understand (n=149)	134 (89.9%)	10 (6.7%)	5 (3.4%)
Information was useful (n=149)	137 (92.0%)	10 (6.7%)	2 (1.3%)
**Physical Activity**			
Information easy to understand (n=116)	109 (93.9%)	6 (5.2%)	1 (0.9%)
Information was useful	111 (95.7%)	4 (3.4%)	1 (0.9%)

For peer review only - http://bmjopen.bmj.com/site/about/guidelines.xhtml

(n=116)			
**Smoking cessation**			
Information easy to understand (n=42)	41 (97.6%)	1 (2.4%)	0 (0%)
Information was useful (n=42)	41 (97.6%)	0 (0%)	1 (2.4%)

For peer review only

Table 3. Example Text Messages for the CHAT and CHAT-DM Studies

CHAT TEXT [6 texts/wk]		CHAT-DM TEXT [6 texts/wk]
**General Education (CVD) [2x/wk]*** The most common risk factors for coronary artery disease are smoking, obesity, high blood pressure, high cholesterol and diabetes. However, most of them can be controlled in an appropriate way.		**General Education (DM) [1x/wk]** Diabetes is not terrible and there are many things you can do to prevent problems from diabetes, such as monitoring blood glucose, watching your diet, keeping fit, and taking pills regularly.
**Blood Pressure Control [2x/wk]** Most people do not experience any symptoms of high blood pressure. Do not stop taking blood pressure medication unless directed by your doctor. It is important for patients with hypertension to take medication diligently and to monitor their blood pressure on a regular basis.		**Blood Pressure Control [1x/wk]** Home Blood Pressure Monitoring is highly recommended! You can get an accurate picture of your heart health and understand daily changes in blood pressure, which is helpful for doctors to adjust medications for you.
**Medication Adherence [1x/wk]**		**Medication Adherence [1x/wk]**

For peer review only - http://bmjopen.bmj.com/site/about/guidelines.xhtml

Do you have a problem remembering to take your blood pressure medications? If so, try to tell your family about your medicine schedule so they can remind you.		Talk to doctors about your concerns and any uncomfortable symptoms after taking pills. Let your doctor help you to find the right medication for you.
**Physical Activity [1x/wk]**<br>You can still choose low-intensity exercise even after heart attack, such as walking and Tai Chi, at a slower pace and stick to your exercise plan. Always consult your physician before beginning any exercise program.		**Physical Activity [1x/wk]**<br>Try brisk walking – a convenient, safe and cost-effective way of exercising! It's good for your heart and will help control blood glucose.
**Smoking Cessation [1x/wk]**[†]<br>Do you worry about your family having health problems because of your smoking? Quitting is an important choice you can make to benefit your family's health, too. Secondhand smoke can cause respiratory disease, lung cancer, and heart disease.		**Diabetes Management [1x/wk]**<br>See a doctor before you travel. Always carry your diabetes medications and insulin, glucose meter, and strips, so that you can better monitor your glucose. Carry some hard candy and crackers to avoid low sugar.

For peer review only - http://bmjopen.bmj.com/site/about/guidelines.xhtml

		**Life style intervention [1x/wk]**<br>Individuals with diabetes should consume a balanced diet, and eat smaller but more frequent meals. Consider splitting your meal and save it for a snack later. Some healthy snack choices include tomatoes, cucumbers, and sugar-free biscuits.

*** In CHAT, non-smokers receive two General Education messages per week, while smokers only receive one per week.**

† In CHAT, only smokers receive smoking cessation messages.

For peer review only

Table 4. Baseline and follow-up data collection

Information	Baseline	6 month
Basic and contact information	√	√
Physical examination: BP, HR, waist circumference, weight, height	√	√
Ambulatory blood pressure	√	√
Outcome	√	√
Hospitalizations	√	√
Current medications	√	√
Medication adherence (Morisky)	√	√
Physical activity (IPAQ)	√	√
CVD functional status (SAQ)	√	√
Health status (EQ-5D)	√	√
Socioeconomic status	√	√
Risk factors control	√	√
Urine cotinine/nicotine test	√	√
Blood urine sample for core lab and local test	√	√

For peer review only - http://bmjopen.bmj.com/site/about/guidelines.xhtml

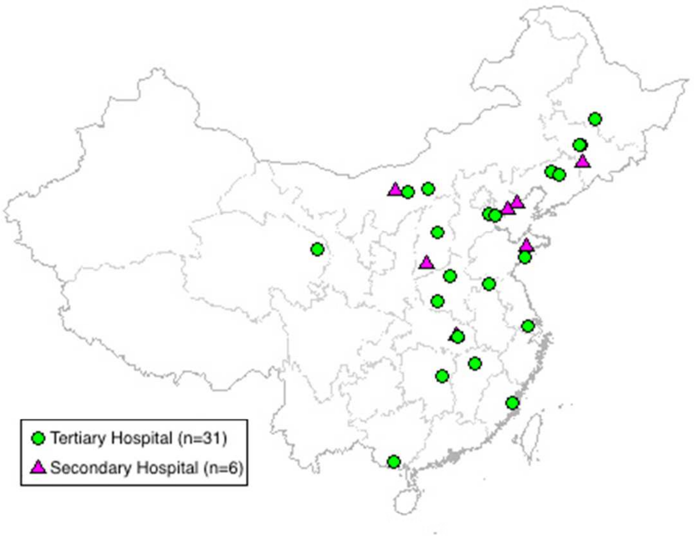

Figure 1. Geographic distribution of sites in CHAT and CHAT-DM studies

169x111mm (96 x 96 DPI)

view only

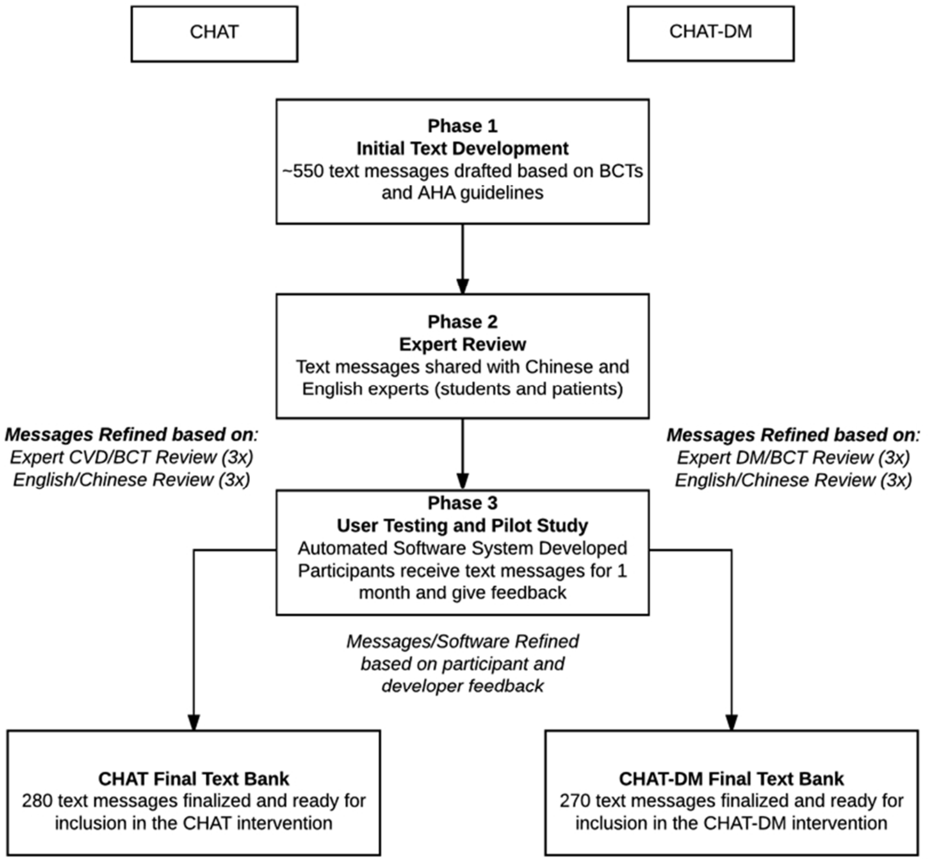

Figure 2. Text Development Process

Supplementary Materials

Design and rationale of the Cardiovascular Health and Text Messaging (CHAT) Study and CHAT-Diabetes Mellitus (CHAT-DM) Study: two randomized controlled trials of text messaging to improve secondary prevention for coronary heart disease and diabetes

TABLE OF CONTENTS

Page 2-3: CHAT and CHAT-DM studies site investigators by hospital

For peer review only

CHAT and CHAT-DM studies site investigators by hospital

1. China-Japan Union Hospital of Jilin University, Ping Yang, Zhaohui Feng, Mei Ding, Bing Li;
2. Dongfeng County Hospital, Wei Liu, Xiaoxin Li, Yanbin Zhang, Wen Cui, Sen Shi;
3. First Hospital of Shanxi Medical University, Qinghua Han, Liqin Duan, Chunrong Jin;
4. General Hospital of China FAW Group Corporation, Hongtao Pan, Lei Sun, Peng Chen;
5. Inner Mongolia Baogang Hopital, Yongdong Li, Liqing Jia, Xingxing Ren;
6. Jiangxi Provincial People's Hospital, Lang Hong, Ji Hong, Linfeng Li, Lihua Yuan, Yun Li;
7. Laixi People's Hospital, Xu Jiang, Hui Liu ,Guohao Xu, Yanrong Song;
8. Laoting County Hospital, Keyong Shang, Changjiang Liu, Kuituan Xi, Ying Yu;
9. Lujiang County People's Hospital, Kelian Xu, Cunqi Wang, Yongan Qian;
10. Nanyang Central Hospital, Shouzhong Yang, Yudong Li, Jianbu Gao, Songyu Zhang;
11. Port Hospital of Hebei Port Group Company, Ltd, Xia Wu, Penghui Yang, Xueqing Wang;
12. Qingdao Fuwai Hospital, Xianyan Jiang, Bin Zhang, Yumei Dong, Cheng Zheng, Wenchuan Hu;
13. Qinghai Cardiovascular and Cerebrovascular Hospital, Huiping Bian, Bo Chen, Xiaojuan Han, Na Han;
14. Qinzhou Second People's Hospital, Liyuan Chen, Qiuxia Liu, Lin Chen;
15. Quwo County People's Hospital, Xiwu Wang, Zhenlin Wu, Qiang Wang, Zhaohui Shangguan;
16. Shanxi Cardiovascular Hospital, Bin Yang, Yingting Yang;
17. Shenyang the Fourth Hospital of People, Yinjun Li, Wei Jiang;
18. Shuangcheng People's Hospital, Cheng Zhang, Aiguo Sun, Xuemei Wang;
19. Suzhou Kowloon Hospital, Shanghai Jiao Tong University School of Medicine, Feng Liu, Qiaoxia Zhou, Bingbing Zhu, Jie Guo;
20. The Affiliated Hospital of Qingdao University, Changyong Zhou, Yini Wang, Tao Yu, Zhe Su;
21. The First Affiliated Hospital of Fujian Medical University, Jinxiu Lin, Dajun Chai, Qinghui Guo;
22. The Affiliated Hospital of Xuzhou Medical University, Dongye Li, Yuanyuan Luo, Junhong Chen, Wei Qian, Qian Yu, Lulu Sun;
23. The First Hospital of Jilin University, Yang Zheng, Zhaoxi Liu, Lin Zou;
24. The Fourth Affiliated Hospital of China Medical University, Yuanzhe Jin, Xiaohong Zhang, Weina Hu;
25. The people's hospital of Dongxihu district of Wuhan ,Yongbo Lin, Ling Zhou, Hanliang Dan;
26. The People's Hospital of Liaoning Province, Zhanquan Li, Ying Liu, Dan Li;

For peer review only

27. The Second Affiliated Hospital of Xuzhou Medical College, Weiheng Wu, Li Li, Sai Zhang;
28. The Second Affiliated Hospital of Zhengzhou University, Xianen Fa, Lihua Zhang, Liqiang Sun, Youxu Jiang;
29. Tianjin Medical University General Hospital, Yuemin Sun, Bo Bian;
30. TEDA International Cardiovascular Hospital, Zhigang Liu, Zhipeng Guo, Cun Zhang;
31. Tongji Hospital of Tongji Medical College, Huazhong University of Science and Technology, Jiangang Jiang, Xiaoqing Shen, Ting Yu;
32. Wuhan Asia Heart Hospital, Xi Su, Songzhi Zhao, Wei Wu, Yujing Fan;
33. Wulate County People's Hospital, Jinlan Xu, Lei Xia, Yunmei Wang;
34. Xiangtan Central Hospital, He Huang, Jianping Zeng, Mingxing Wu, Yi Zhou;
35. Xinmin People's Hospital, Bo Jiang, Liwei Qi, Tongying Li;
36. Xuzhou First People's Hospital, Hongju Zhang, Chen Bian, Wei Li;
37. Zhengzhou Central Hospital, Lin Zhang, Yumei Guo;

For peer review only

SPIRIT
STANDARD PROTOCOL ITEMS: RECOMMENDATIONS FOR INTERVENTIONAL TRIALS

SPIRIT 2013 Checklist: Recommended items to address in a clinical trial protocol and related documents*

Section/item	Item No	Description	Addressed on page number
**Administrative information**			
Title	1	Descriptive title identifying the study design, population, interventions, and, if applicable, trial acronym	_____1_______
Trial registration	2a	Trial identifier and registry name. If not yet registered, name of intended registry	_____3_______
	2b	All items from the World Health Organization Trial Registration Data Set	____ N/A _____
Protocol version	3	Date and version identifier	____ N/A _____
Funding	4	Sources and types of financial, material, and other support	_____21______
Roles and responsibilities	5a	Names, affiliations, and roles of protocol contributors	____ 1-2______
	5b	Name and contact information for the trial sponsor	_____21______
	5c	Role of study sponsor and funders, if any, in study design; collection, management, analysis, and interpretation of data; writing of the report; and the decision to submit the report for publication, including whether they will have ultimate authority over any of these activities	_____6, 22____
	5d	Composition, roles, and responsibilities of the coordinating centre, steering committee, endpoint adjudication committee, data management team, and other individuals or groups overseeing the trial, if applicable (see Item 21a for data monitoring committee)	_____22______

For peer review only - http://bmjopen.bmj.com/site/about/guidelines.xhtml

Introduction

Background and rationale	6a	Description of research question and justification for undertaking the trial, including summary of relevant studies (published and unpublished) examining benefits and harms for each intervention	_____4-6_____
	6b	Explanation for choice of comparators	______8______
Objectives	7	Specific objectives or hypotheses	_____5-6_____
Trial design	8	Description of trial design including type of trial (eg, parallel group, crossover, factorial, single group), allocation ratio, and framework (eg, superiority, equivalence, noninferiority, exploratory)	______6______

Methods: Participants, interventions, and outcomes

Study setting	9	Description of study settings (eg, community clinic, academic hospital) and list of countries where data will be collected. Reference to where list of study sites can be obtained	______6______
Eligibility criteria	10	Inclusion and exclusion criteria for participants. If applicable, eligibility criteria for study centres and individuals who will perform the interventions (eg, surgeons, psychotherapists)	______7______
Interventions	11a	Interventions for each group with sufficient detail to allow replication, including how and when they will be administered	_____7-12____
	11b	Criteria for discontinuing or modifying allocated interventions for a given trial participant (eg, drug dose change in response to harms, participant request, or improving/worsening disease)	______8______
	11c	Strategies to improve adherence to intervention protocols, and any procedures for monitoring adherence (eg, drug tablet return, laboratory tests)	_____12______
	11d	Relevant concomitant care and interventions that are permitted or prohibited during the trial	______8______
Outcomes	12	Primary, secondary, and other outcomes, including the specific measurement variable (eg, systolic blood pressure), analysis metric (eg, change from baseline, final value, time to event), method of aggregation (eg, median, proportion), and time point for each outcome. Explanation of the clinical relevance of chosen efficacy and harm outcomes is strongly recommended	____14-15____
Participant timeline	13	Time schedule of enrolment, interventions (including any run-ins and washouts), assessments, and visits for participants. A schematic diagram is highly recommended (see Figure)	______6______

For peer review only - http://bmjopen.bmj.com/site/about/guidelines.xhtml

Sample size	14	Estimated number of participants needed to achieve study objectives and how it was determined, including clinical and statistical assumptions supporting any sample size calculations	____15-16____
Recruitment	15	Strategies for achieving adequate participant enrolment to reach target sample size	______6______
**Methods: Assignment of interventions (for controlled trials)**			
Allocation:			
Sequence generation	16a	Method of generating the allocation sequence (eg, computer-generated random numbers), and list of any factors for stratification. To reduce predictability of a random sequence, details of any planned restriction (eg, blocking) should be provided in a separate document that is unavailable to those who enrol participants or assign interventions	______7______
Allocation concealment mechanism	16b	Mechanism of implementing the allocation sequence (eg, central telephone; sequentially numbered, opaque, sealed envelopes), describing any steps to conceal the sequence until interventions are assigned	______7______
Implementation	16c	Who will generate the allocation sequence, who will enrol participants, and who will assign participants to interventions	______7______
Blinding (masking)	17a	Who will be blinded after assignment to interventions (eg, trial participants, care providers, outcome assessors, data analysts), and how	______7______
	17b	If blinded, circumstances under which unblinding is permissible, and procedure for revealing a participant's allocated intervention during the trial	____ N/A _____
**Methods: Data collection, management, and analysis**			
Data collection methods	18a	Plans for assessment and collection of outcome, baseline, and other trial data, including any related processes to promote data quality (eg, duplicate measurements, training of assessors) and a description of study instruments (eg, questionnaires, laboratory tests) along with their reliability and validity, if known. Reference to where data collection forms can be found, if not in the protocol	____12-13____
	18b	Plans to promote participant retention and complete follow-up, including list of any outcome data to be collected for participants who discontinue or deviate from intervention protocols	____ N/A _____

For peer review only - http://bmjopen.bmj.com/site/about/guidelines.xhtml

Data management	19	Plans for data entry, coding, security, and storage, including any related processes to promote data quality (eg, double data entry; range checks for data values). Reference to where details of data management procedures can be found, if not in the protocol	____13-14____
Statistical methods	20a	Statistical methods for analysing primary and secondary outcomes. Reference to where other details of the statistical analysis plan can be found, if not in the protocol	____15-16____
	20b	Methods for any additional analyses (eg, subgroup and adjusted analyses)	_____16______
	20c	Definition of analysis population relating to protocol non-adherence (eg, as randomised analysis), and any statistical methods to handle missing data (eg, multiple imputation)	_____15______
**Methods: Monitoring**			
Data monitoring	21a	Composition of data monitoring committee (DMC); summary of its role and reporting structure; statement of whether it is independent from the sponsor and competing interests; and reference to where further details about its charter can be found, if not in the protocol. Alternatively, an explanation of why a DMC is not needed	____ N/A _____
	21b	Description of any interim analyses and stopping guidelines, including who will have access to these interim results and make the final decision to terminate the trial	____ N/A _____
Harms	22	Plans for collecting, assessing, reporting, and managing solicited and spontaneously reported adverse events and other unintended effects of trial interventions or trial conduct	_____13______
Auditing	23	Frequency and procedures for auditing trial conduct, if any, and whether the process will be independent from investigators and the sponsor	____ N/A _____
**Ethics and dissemination**			
Research ethics approval	24	Plans for seeking research ethics committee/institutional review board (REC/IRB) approval	______6______
Protocol amendments	25	Plans for communicating important protocol modifications (eg, changes to eligibility criteria, outcomes, analyses) to relevant parties (eg, investigators, REC/IRBs, trial participants, trial registries, journals, regulators)	____ N/A _____

For peer review only - http://bmjopen.bmj.com/site/about/guidelines.xhtml

Consent or assent	26a	Who will obtain informed consent or assent from potential trial participants or authorised surrogates, and how (see Item 32)	______6______
	26b	Additional consent provisions for collection and use of participant data and biological specimens in ancillary studies, if applicable	______6______
Confidentiality	27	How personal information about potential and enrolled participants will be collected, shared, and maintained in order to protect confidentiality before, during, and after the trial	____13-14____
Declaration of interests	28	Financial and other competing interests for principal investigators for the overall trial and each study site	_____21______
Access to data	29	Statement of who will have access to the final trial dataset, and disclosure of contractual agreements that limit such access for investigators	____12-14____
Ancillary and post-trial care	30	Provisions, if any, for ancillary and post-trial care, and for compensation to those who suffer harm from trial participation	____ N/A _____
Dissemination policy	31a	Plans for investigators and sponsor to communicate trial results to participants, healthcare professionals, the public, and other relevant groups (eg, via publication, reporting in results databases, or other data sharing arrangements), including any publication restrictions	_____20______
	31b	Authorship eligibility guidelines and any intended use of professional writers	_____21______
	31c	Plans, if any, for granting public access to the full protocol, participant-level dataset, and statistical code	_____20______
**Appendices**			
Informed consent materials	32	Model consent form and other related documentation given to participants and authorised surrogates	____ N/A _____
Biological specimens	33	Plans for collection, laboratory evaluation, and storage of biological specimens for genetic or molecular analysis in the current trial and for future use in ancillary studies, if applicable	_____13______

*It is strongly recommended that this checklist be read in conjunction with the SPIRIT 2013 Explanation & Elaboration for important clarification on the items. Amendments to the protocol should be tracked and dated. The SPIRIT checklist is copyrighted by the SPIRIT Group under the Creative Commons "Attribution-NonCommercial-NoDerivs 3.0 Unported" license.

For peer review only - http://bmjopen.bmj.com/site/about/guidelines.xhtml

BMJ Open

Design and rationale of the Cardiovascular Health and Text Messaging (CHAT) Study and CHAT-Diabetes Mellitus (CHAT-DM) Study: two randomized controlled trials of text messaging to improve secondary prevention for coronary heart disease and diabetes

Journal:	*BMJ Open*
Manuscript ID	bmjopen-2017-018302.R1
Article Type:	Protocol
Date Submitted by the Author:	13-Sep-2017
Complete List of Authors:	Huo, Xiqian; Fuwai Hospital, National Center for Cardiovascular Diseases, State Key Laboratory of Cardiovascular Disease<br>Spatz, Erica; Yale University<br>Ding, Qinglan; Yale University<br>Horak, Paul ; Yale University<br>Zheng, Xin; Fuwai Hospital, National Center for Cardiovascular Diseases, State Key Laboratory of Cardiovascular Disease<br>Masters, Claire ; Yale University<br>Zhang, Haibo; Cardiovascular Institute & Fu Wai Hospital, Chinese Academy of Medical Sciences & Peking Union Medical College, China Oxford Centre for International Health Research<br>Irwin, Melinda; Yale School of Public Health<br>Yan, Xiaofang; Fuwai Hospital, National Center for Cardiovascular Diseases, State Key Laboratory of Cardiovascular Disease<br>Guan, Wenchi; Cardiovascular Institute & Fu Wai Hospital, Chinese Academy of Medical Sciences & Peking Union Medical College, China Oxford Centre for International Health Research,<br>Li, Jing; Cardiovascular Institute & Fu Wai Hospital, Chinese Academy of Medical Sciences & Peking Union Medical College, China Oxford Centre for International Health Research<br>Li, Xi; Fuwai Hospital, National Center for Cardiovascular Diseases, State Key Laboratory of Cardiovascular Disease<br>Spertus, John; Mid America Heart Institute of Saint Luke's Hospital<br>Masoudi, Frederick; Division of Cardiology, University of Colorado Anschutz Medical Campus<br>Krumholz, Harlan; Yale School of Public Health<br>Jiang, Lixin; Fuwai Hospital, National Center for Cardiovascular Diseases, State Key Laboratory of Cardiovascular Disease
**Primary Subject Heading**:	Research methods
Secondary Subject Heading:	Research methods, Medical management
Keywords:	Coronary heart disease < CARDIOLOGY, diabetes, text messaging, behavioral intervention, secondary prevention

For peer review only

Design and rationale of the Cardiovascular Health and Text Messaging (CHAT) Study and CHAT-Diabetes Mellitus (CHAT-DM) Study: two randomized controlled trials of text messaging to improve secondary prevention for coronary heart disease and diabetes

Xiqian Huo, MD[*]; Erica S. Spatz, MD, MHS[*]; Qinglan Ding, MS; Paul Horak, BS; Xin Zheng, MD, PhD; Claire Masters, MHP; Haibo Zhang, MD; Melinda Irwin, PhD, MPH; Xiaofang Yan, MD; Wenchi Guan, MD; Jing Li, MD, PhD; Xi Li, MD, PhD; John A. Spertus, MD, MPH; Frederick A. Masoudi, MD, MSPH; Harlan M. Krumholz, MD, SM[**]; Lixin Jiang, MD, PhD[**]

(* Joint first authors)

(** Joint senior authors)

China Oxford Center for International Health Research, Fuwai Hospital, National Center for Cardiovascular Diseases, Chinese Academy of Medical Sciences and Peking Union Medical College, Beijing, People's Republic of China (X.H, X.Z, H.Z, X.Y, W.G, J.L, X.L, L.J);

Center for Outcomes Research and Evaluation, Yale University/Yale-New Haven Hospital, New Haven, Connecticut, USA (E.S.S, Q.D, P.H, C.M, M.I, H.M.K);

Health Outcomes Research, Saint Luke’s Mid America Heart Institute/University of Missouri-Kansas City, Kansas City, Missouri, USA (J.A.S);

For peer review only

For peer review only - http://bmjopen.bmj.com/site/about/guidelines.xhtml

Division of Cardiology, University of Colorado Anschutz Medical Campus, Aurora, Colorado, USA (F.A.M).

Correspondence: Lixin Jiang, MD, PhD, National Clinical Research Center of Cardiovascular Diseases, Fuwai Hospital, National Center for Cardiovascular Diseases, 167 Beilishi Road, Xicheng District, Beijing, 100037, People's Republic of China. Tel: +86 10 8839 6203; Fax: +86 10 8836 5201. Email: jiangl@fwoxford.org

For peer review only

ABSTRACT

Introduction: Mobile health interventions have the potential to promote risk factor management and lifestyle modification, and are a particularly attractive approach for scaling across healthcare systems with limited resources. We are conducting two randomized trials to evaluate the efficacy of text-based health messages in improving secondary coronary heart disease (CHD) prevention among patients with or without diabetes.

Methods and analysis: The Cardiovascular Health And Text messaging (CHAT) and CHAT-Diabetes Mellitus (CHAT-DM) Studies are multi-center, single-blind, randomized controlled trials of text messaging versus standard treatment with 6 months of follow-up conducted in 37 hospitals throughout 17 provinces in China. The intervention group receives 6 text messages per week which target blood pressure control, medication adherence, physical activity, smoking cessation (when appropriate), glucose monitoring and lifestyle

For peer review only - http://bmjopen.bmj.com/site/about/guidelines.xhtml

recommendations including diet (in CHAT-DM). The text messages were developed based on behavioral change techniques, using models such as information-motivation-behavioral skills model, goal-setting and provision of social support. The estimated sample size is 790 in CHAT Study and 480 in CHAT-DM Study. In CHAT, the primary outcome is the change in systolic blood pressure (SBP) at 6 months. Secondary outcomes include a change in proportion of patients achieving a SBP<140mm Hg, low-density lipoprotein cholesterol (LDL-C), physical activity, medication adherence, body mass index (BMI), and smoking cessation. In CHAT-DM, the primary outcome is the change in glycemic hemoglobin (HbA_{1C}) at 6 months. Secondary outcomes include a change in proportion of patients achieving HbA_{1C}<7%, fasting blood glucose, SBP, LDL-C, BMI, physical activity and medication adherence.

Ethics and dissemination: The central ethics committee at the China National Center for Cardiovascular Disease (NCCD) approved the CHAT and CHAT-DM studies. Results will be disseminated via usual scientific forums including peer-reviewed publications.

Trial registration number: CHAT (NCT02888769) and CHAT-DM (NCT02883842); pre-results.

KEYWORDS: coronary heart disease, diabetes, text messaging, behavioral intervention, secondary prevention

For peer review only - http://bmjopen.bmj.com/site/about/guidelines.xhtml

Strengths and Limitations of study:

1. The main strengths of the study are that it evaluates the efficacy of an innovative, simple, scalable and cost-effective intervention for improving secondary prevention of CHD. The trials are the first to investigate the effectiveness of text messages to support management of CHD and DM among a large and diverse population in China, and have the potential for scaling across healthcare systems in resource-constrained settings.
2. The study addressed the role of text messages in managing multiple risk factors in patients with CHD or DM. Moreover, there is currently little evidence about the effectiveness of such interventions for high-risk patients with coronary heart disease and diabetes.
3. The CHAT and CHAT-DM studies are further distinguished by their relative large sample sizes and culturally appropriate, theory-driven text messages. The messages were developed using behavior change technique and tailored to a specific Chinese patient population.
4. In contrast to most prior single center text-messaging trials, these two studies are conducted at 37 participating sites spread across a large and geographically diverse country. The results of the studies may be more generalizable.
5. However, medication adherence and physical activity are measured by self-report, which carries the possibility of recall bias and social desirability bias.

For peer review only - http://bmjopen.bmj.com/site/about/guidelines.xhtml

INTRODUCTION

The benefits of secondary prevention strategies for coronary heart disease (CHD) targeting lifestyle modification and risk factor management are well-established worldwide,[1, 2] however adoption of these strategies is suboptimal.[3] Smoking, inactivity and obesity are prevalent among people with established CHD and control of hypertension and diabetes are often suboptimal. Additionally, medication adherence is poor. Prior studies revealed that only three-fourths of patients take all medications from their discharge prescriptions by 120 days after discharge.[4] Furthermore, less than half of patients hospitalized with acute myocardial infarction (AMI) are adherent to evidence-based medications 1 year later, with the greatest gaps in adherence occurring in the first 6 months after treatment initiation.[5-7]

In lower and middle-income countries (LMICs), including China, which face a growing burden of cardiovascular disease and greater challenges to medication access for secondary prevention, over two-thirds of patients with CHD take no medication.[8-10] While high medication costs are a barrier,[11] there is also limited time for education and consultation regarding lifestyle and medication management during clinic visits, which tend to be very brief.[12, 13] Therefore, innovative and cost-effective interventions to enhance adherence are urgently needed.

Mobile phones are pervasive and thus can be used to deliver interventions that help people to adopt secondary prevention strategies for CHD in LMICs. They are already a primary, inexpensive and quick form of communication, and are also widely used to schedule alerts and reminders. In addition, the number of

For peer review only - http://bmjopen.bmj.com/site/about/guidelines.xhtml

mobile phone users has grown exponentially in the past decade worldwide, and is projected to reach 4.77 billion by 2017.[14]. As of August 2016, China had the largest number of mobile phone owners in the world, at 1.3 billion.[15] Mobile phones are also used across all geographic regions and income levels. Due to the ubiquity and convenience, nearly 200,000 mobile phone messages are sent every second in China[16]. Text messaging has the potential to be a scalable and powerful tool to deliver health information.[17, 18]

Prior studies of mobile phone text messaging have been conducted to improve glycemic control,[19] hypertension,[20] medication adherence,[21] as well as to promote smoking cessation,[22] and physical activity.[23, 24] These trials have contributed important knowledge, and some, such as TEXT-ME[25] and TExT-MED[26] suggest that text-messaging interventions can influence patient behaviors and improve risk profiles. Still, several questions remain about the generalizability of these findings, especially for populations from LMICs. Most trials to date have been designed to target a single condition; yet patients with cardiovascular disease usually manage multiple conditions, requiring several lifestyle and treatment recommendations. Additionally, most studies of text messaging interventions have not been grounded in behavioral change techniques (BCT),[27, 28] which may influence the efficacy and generalizability of the intervention. Finally, most prior studies have been limited to single site interventions, many of which were underpowered and/or limited by selection bias. More data are needed to understand whether text-messaging interventions should be adopted as an effective strategy for supporting cardiovascular disease prevention among diverse populations from LMICs.[29]

For peer review only - http://bmjopen.bmj.com/site/about/guidelines.xhtml

Accordingly, we designed and conducted the Cardiovascular Health And Texting (CHAT) and CHAT-Diabetes Mellitus (CHAT-DM) studies. The primary objective of these two studies is to evaluate the efficacy of an automated text message-based intervention, based on behavioral change techniques, in improving risk factor control and adoption of healthy lifestyle behaviors among patients with known CHD, with or without DM, who were discharged from multiple hospitals throughout China.

METHODS AND ANALYSIS

Study Overview

The CHAT and CHAT-DM studies are multi-center, single-blind, 2-arm, randomized controlled trials of an automated mobile phone text message-based intervention with 6 months of follow-up. Patients were recruited from 37 hospitals across 17 provinces in China (Figure 1, supplementary material). The enrollment of participants began on August 16, 2016. The two studies were registered at http://www.clinicaltrials.gov (NCT02888769 and NCT02883842) accordingly. We retrospectively registered the trial 7 days in CHAT and 5 days in CHAT-DM study after enrollment of the first patient, beyond what is recommended by ICMJE that trials registry at or before the time of first patient enrollment, as we referred to the FDA AA801 public law on the Clinicaltrials.gov website that clinical trials are registered no later than 21 days after the first patient was enrolled. The recruitment was completed in April 2017, and the last follow-up visit is expected to finish in October 2017. All participants provided written informed consent at the initial

For peer review only - http://bmjopen.bmj.com/site/about/guidelines.xhtml

trial visit.

Study Population

In CHAT, patients were eligible if they had established CHD defined as having a history of AMI and/or percutaneous coronary intervention (PCI), having access to a mobile phone to read and send text messages, and did not have diabetes. In CHAT-DM, patients were eligible if they had a history of documented CHD (as defined in CHAT) and diabetes, and had access to a mobile phone to read and send text messages. In both studies, patients were excluded if they could not read or send text messages, had cognitive or communication disorders, or could not provide informed consent. A 'screening log' of basic demographic information and reasons for not participating in patients deemed ineligible but who declined to participate has been maintained. We recruited patients who had been hospitalized with CHD, with or without DM, and had medical records with definite discharge diagnoses available. The diagnoses of CHD and DM given by local physicians were adjudicated centrally, based upon review of the patients' medical charts, which were sent to the China National Center for Cardiovascular Disease (NCCD).

Randomization and blinding

Participants were randomly allocated to either the intervention or control arms in a 1:1 ratio using a computerized randomization system. In order to achieve a balance of participants' characteristics in both arms, we employed a stratified

For peer review only - http://bmjopen.bmj.com/site/about/guidelines.xhtml

randomization approach, based on age, gender, AMI history, education degree and medical insurance type within each study. Researchers, statisticians and clinic staff were blinded to treatment allocation.

Trial intervention

Participants in the intervention groups of the CHAT and CHAT-DM studies receive text messages about CHD risk factor modification for 6 months as well as standard treatment (described below). The control group in both studies receive two thank-you text messages without risk factor modification support each month as well as standard treatment. A training session was held by research staff upon enrollment to ensure that all participants were capable of receiving, reading and sending text messages on their mobile phones. Prior to commencement, a test message was sent to each participant to confirm the phone number and that the system was working effectively. Whether the test messages had reached the patients would be recorded by local study staff, and if patients did not receive messages, local study staff would confirm and update the correct phone number. Participants were also informed that they could withdraw from the study by responding to a text message with a specific character. Researchers at NCCD could monitor the status of text message delivery, review responses sent by participants and manage withdrawal via a customized software platform. For instance, logs were kept to assess the proportion of text messages successfully delivered, the exact time of messages being sent and the reasons for those that failed to be sent.

For peer review only - http://bmjopen.bmj.com/site/about/guidelines.xhtml

CHAT and CHAT-DM intervention development

A bank of 550 text messages (280 in CHAT and 270 in CHAT-DM) was developed by a multidisciplinary team of cardiologists, endocrinologists, psychologists, nurses and public health researchers using a three-phase systematic and iterative approach (Figure 2). In the CHAT study, text messages are categorized into five groups: (1) general education on CHD and AMI, (2) medication adherence, (3) blood pressure control, (4) physical activity, and (5) smoking cessation. The text messages in the CHAT-DM study cover a range of diabetes self-management topics including: (1) general education on CHD and DM, (2) medication adherence, (3) glucose monitoring and control, (4) blood pressure control, (5) physical activity, and (6) lifestyle recommendations such as diet and foot care. All text messages are in Chinese, and each text message is less than 70 Chinese characters, which would be equivalent to a 140-160-character message in English.

Phase 1 Approach to developing text messages

Text messages were originally drafted by members of the research team in Chinese based on current guidelines[30-32] and standards of care pertaining to cardiovascular health and diabetes care. All messages were grounded in behavioral change techniques previously used to develop short health messages, providing information, goal-setting, motivation, social support and stress management advice.[33] In order to strengthening the designing and reporting of theory-based BCT interventions, we took into account the two recent BCT taxonomies by Michie. S, and his colleagues.[34, 35] Efforts were devoted to selecting the behavioral change techniques most applicable to the

For peer review only - http://bmjopen.bmj.com/site/about/guidelines.xhtml

Chinese cultural context and compatible with Chinese beliefs and values (Table 1). For example, some text messages applied traditional Chinese aphorisms, chengyu, and catchy rhyme schemes in order to make them more acceptable to patients. As another example, in designing text messages that were motivating, especially in challenging situations, the multidisciplinary group felt that Chinese people tend to prefer more direct and structured counseling instructions rather than indirect and insight-oriented approaches (a category of psychotherapeutic approaches that promote individuals' adaptive behavior and course of action in life through understanding their internal motivation);[36] as such, text messages were written to provide practical approaches and real-life examples instead of abstract theories.[37, 38] Social and family-oriented goals were used more often than individual achievement to help improve health behaviors, consistent with cultural norms in China.[39, 40] These messages were sent to experts in behavioral change techniques and counseling for review and were used as examples to draft the initial bank of 550 text messages. Once drafted, all messages were reviewed, critiqued and revised within the internal research team.

Phase 2 Expert review

An expert panel made up of clinicians and academics reviewed each round of text message drafts with a different focus in each iteration. First, clinical experts (cardiologists, endocrinologists and psychologists) considered the accuracy, clarity and practical usefulness of each text message. Second, 21 messages from all categories in the CHAT and CHAT-DM studies text banks were randomly selected and translated into English by bilingual researchers. Experts in the fields of cardiology, endocrinology, epidemiology, psychology

For peer review only - http://bmjopen.bmj.com/site/about/guidelines.xhtml

and behavioral science in the U.S. reviewed them and provided suggestions for further refinement. Finally, Chinese researchers and a linguist reviewed all 550 text messages, further refined the language, and paid special attention to the cultural meaning of all text messages to ensure better understanding among the targeted population (elderly Chinese patients with cardiovascular disease, with and without diabetes). Once feedback from the experts was addressed, the text bank was updated and prepared for user testing.

Phase 3 User testing and pilot study

For user testing, 19 randomly selected text messages from the finalized text bank in the CHAT and CHAT-DM studies were distributed to 39 individuals with CHD (with or without diabetes) for feedback. Likert-type items in a survey were used to assess usefulness and ease of understanding of the text messages. Open-ended questions were asked to obtain suggestions for improvement from participants. In total, 92.4% (582/630) rated the messages as easy to understand and 93.2% (587/630) rated the messages as useful. All 550 text messages were further modified based on feedback from user testing. Results of the scores for the message categories in this phase are summarized in Table 2.

Next, the refined text message banks underwent pilot testing to evaluate the efficacy of the message delivery system and quality of the user experience. A customized software program sent messages through a gateway interface, allowing them to be sent to all individuals' mobile phones free of charge and at a bulk-rate cost (0.1 yuan per message, or .01 USD) to the research team. Participants from the user testing were enrolled in the pilot testing after

For peer review only - http://bmjopen.bmj.com/site/about/guidelines.xhtml

providing written informed consent (n=33). For the pilot study, messages drawn from the entire text bank were sent to participants. At the end of the 1-month pilot study, 30 participants reported their experience of receiving messages and commented on frequency, timing, content and potential impact of text messages. Minor changes were made to the text message bank following the pilot study based on this feedback.

Frequency and timing of text message delivery

Each participant in the intervention group of the CHAT and CHAT-DM studies receives 6 text messages per week, randomly selected by the software system, across a 6 month timeframe. The messages are sent at one of three random times (9am, 12pm or 4pm), during weekends and weekdays (excluding Monday to give people a break). The text messages were designed to be semi-personalized with participant's preferred name at beginning of some messages, as well as considering participant's smoking status. In the CHAT study, participants receive two general education messages on CHD (one if an active smoker), two blood pressure control messages, one medication adherence message, and one physical activity message per week. Smokers receive one smoking cessation message per week. Participants in the CHAT-DM intervention group receive one general message, one blood pressure related message, one glucose control message, one lifestyle modification message, one medication adherence message and one physical activity message per week (Table 3).

Initially, a test message was sent to each participant to ensure that the correct mobile phone number had been recorded and the system was functioning

For peer review only - http://bmjopen.bmj.com/site/about/guidelines.xhtml

appropriately. All participants received a personalized welcome message, a follow-up reminder and a birthday greeting while enrolled in the study. Most of the text messages were developed to be unidirectional and participants are not anticipated to reply, however bidirectional text messages, checking on medication adherence and blood pressure/glucose level measurements, are sent at weekly intervals to evaluate patient engagement. Throughout the 6-month follow-up, research staff call participants if they do not respond for two consecutive weeks, and only one call is made per patient during the intervention period, so as not to confound the intervention.

Procedures for Data Collection and Management

Data Collection

Basic and contact information (ID number, address, and phone number), anthropometric data (waist circumference, height and weight), resting blood pressure, heart rate, ambulatory blood pressure, and information on socioeconomic status, risk factor control and current medications were collected at baseline at hospital recruitment sites. Detailed information on patient outcomes (hospitalizations, discharge diagnoses, etc.) was also collected during the survey and hospital records or death certificates obtained where necessary for adjudication. Additional assessments of baseline medication adherence (Morisky Medication Adherence Scales: MMAS-8),[41] physical activity (International Physical Activity Questionnaire: IPAQ),[42, 43] CVD-specific health status (Seattle Angina Questionnaire: SAQ),[44] health status (EuroQol five-dimensional questionnaire: EQ-5D),[45] and smoking status

For peer review only - http://bmjopen.bmj.com/site/about/guidelines.xhtml

were also conducted in person. Lastly, blood and urine samples were collected for local lab tests and eventual transfer to the core lab in Beijing at the biobank of NCCD. To ensure the standardization and accuracy of the sample analysis results, we developed standard operating procedures and trained local study researchers repeatedly regarding samples collection, separation, storage and transfer process. Blood tests, including low-density lipoprotein cholesterol (LDL-C), glycemic hemoglobin (HbA_{1C}) and fasting blood glucose (FBG) will be analyzed at central laboratory. Research staff would collect the above information again as listed in Table 4 at follow-up visit. We conducted on-site monitoring of recruitment, physical measurements, sample collection and document completeness (e.g. informed consent) by trained staff from NCCD to ensure the quality of data collection.

Data Management

A proprietary software platform was developed by the study IT team for use in sending text messages to participants. The platform is capable of sending tailored and semi-personalized text messages to participants and also recording responses. Additionally, this web-based platform is used to monitor project progress, as well as provide management support for hospitals, staff members, equipment and sampling collection and transfer. Trained medical staff members fill out pre-designed on-screen case report forms at each site, and data is then securely transmitted to the central server through automatic electronic transfer. To ensure the reliability and validity of the data, continuous checks are run to ensure that data being entered are complete and meet predefined data formats and ranges. The database is regularly backed-up and

For peer review only - http://bmjopen.bmj.com/site/about/guidelines.xhtml

password protected so that only a limited number of approved staff members can access the data. In order to ensure the confidentiality of all personal information, data confidentiality policies of the NCCD on data collection, storage and analysis have been strictly imposed.

Outcomes

In the CHAT study, the primary outcome is the change in systolic blood pressure (SBP) after 6 months. Secondary outcomes include a change in proportion of patients achieving a SBP<140mm Hg, change in proportion of non-smokers, change in medication adherence categorized by Morisky scale, as well as change in plasma mean level of LDL-C, change in level of body mass index (BMI) and change in level of physical activity. Exploratory outcomes include the prognosis of the patients at 6 months, such as death, non-fatal myocardial infarction, stroke and any re-hospitalization, as well as health status measured by SAQ and EQ-5D.

In the CHAT-DM study, the primary outcome is the change in glycemic hemoglobin (HbA_{1C}) as measured by central blood sample. Secondary outcomes include a change in proportion of patients achieving HbA_{1C}<7%, change in medication adherence, as well as change in mean level of FBG, SBP, LDL-C, BMI and physical activity. Exploratory outcomes include prognosis of patients at 6 months, including death, nonfatal myocardial infarction, stroke and any re-hospitalization, as well as health status (SAQ and EQ-5D).

For peer review only - http://bmjopen.bmj.com/site/about/guidelines.xhtml

Blood pressure is measured on the right upper arm after 5 minutes of rest in a seated position using an electronic blood pressure monitor (Omron HEM-7111; Omron Corporation, Dalian, China). Two measurements are taken and the mean value is calculated. If the difference between the two SBP or diastolic blood pressure (DBP) readings is larger than 5 mm Hg, a third measurement is done, and the mean value of the last two readings is calculated. HbA_{1C} is determined using a high-performance liquid chromatography technique with ADAMS^{TM}A_{1C}HA-8180 (ARKRAY, Inc, Japan). BMI is calculated by dividing weight in kilograms by height in meters squared. Physical activity is measured in metabolic equivalents of task (METs) per minute per week, using the short version of the IPAQ.[42, 43] Medication is assessed via MMAS-8, which scores the adherence from 0 to 8 points.[41] Smoking status is determined using either self-reported smoking status or urine-cotinine testing strip with a cut-off of 200ng/mL cotinine (COT Cotinine Test Colloidal Gold; Hangzhou Clongene Biotech Co., China).[46, 47] Quality of life is measured using Short Version of SAQ[44] and EQ-5D.[45] Local study staff obtained information of baseline hospitalization, readmissions to hospitals and death during the patient's interview, with medical records, death certificates or death records collected as supporting documents. If the patient died at home without any evidentiary material, a structured summary of death conversation with family members would be reported. All information was sent to the NCCD for central adjudication according to pre-specified criteria by trained clinicians. If the patient was re-hospitalized in other hospitals, for example, the study investigators will contact the specific hospital, copy those medical records and transmit them to NCCD as required.

For peer review only - http://bmjopen.bmj.com/site/about/guidelines.xhtml

Statistical Analysis

Intervention evaluation will be carried out on an intention-to-treat (ITT) basis. Values of analyzed parameters at baseline between intervention group and control group will be compared using Student's t-tests for continuous variables or Chi-square tests for categorical variables according to the analysis plan. Mann-Whitney U tests will be used where continuous data are not normally distributed. The primary analysis will employ analysis of covariance (ANCOVA) with baseline values of the analyzed endpoints used as covariates when appropriate. The mean level of each risk factor will also be compared between groups in terms of relative risks, 95% CIs and two-sided p values. We will conduct a pre-specified 2nd analysis with adjustment for patient characteristics as well as subgroup analyses based on age, sex, education, smoking status and tertiles level of endpoints.

All sample size calculations in both studies are for 80% power and 0.05 (one-sided) level of significance, allowing for 20% dropout rate during follow up. In the CHAT study, we estimate a mean SBP level of 132mm Hg [SD, 18mm Hg] in the study population according to the report of previous studies,[48] and assume an absolute reduction in SBP of 5 mm Hg at 6 months from baseline. A total sample size of 800 patients would be adequate to detect this difference in SBP between two groups even when considering the potential compliance to the intervention. In the CHAT-DM study, we assume a mean HbA_{1C} level of 7.2% [SD, 1.6%] based on data from studies involving similar populations[49, 50] and supposed a 0.5% absolute decrease in HbA_{1C} across treatment group. A

For peer review only - http://bmjopen.bmj.com/site/about/guidelines.xhtml

total sample size of 500 patients would be adequate to detect this difference in HbA_{1C} between two groups even when considering the potential compliance to the intervention. The trial results will be reported in accordance with the SPIRIT checklists.

ETHICS AND DISSEMINATION

The findings of the study will be disseminated by standard scientific forums, including peer-review publications and presentations at conferences. The central ethics committee at the NCCD approved the CHAT and CHAT-DM studies. All collaborating hospitals accepted the central ethics approval except for 8, which obtained local approval by internal ethics committees. The Chinese government, which provides financial support, has no role in the design or conduct of the study, the collection, management, analysis, and interpretation of the data; or in the preparation or approval of articles resulting from the studies.

DISCUSSION

The CHAT and CHAT-DM studies aim to assess the efficacy of an innovative intervention for improving secondary prevention of CHD by using simple and cost-effective text-messaging technology among patients with known CHD, with or without DM, throughout China. To the best of our knowledge, these two trials are the first to investigate the efficacy of text messages to support management of CHD and DM among a large population in China, and may set

For peer review only - http://bmjopen.bmj.com/site/about/guidelines.xhtml

For peer review only

a model of such interventions that can be leveraged to improve risk factor management in resource-constrained settings.

The CHAT and CHAT-DM studies have several strengths. While prior studies evaluated the effectiveness of mobile phone text messaging to improve single individual health behaviors, very few trials have addressed the role of text messages in managing multiple risk factors in patients with CHD or DM, which is a reality for many patients. Patients with CHD and DM often have multiple risk factors, yet the care for these conditions is often fragmented. Targeting multiple risk factors concurrently instead of managing a single risk factor may be more efficient and impactful on disease management as this strategy is more patient-centered than disease-centered, and have a greater likelihood of improving risk factor control and cardiovascular outcomes.[51, 52] Additionally, there is currently little evidence about the effectiveness of such interventions for high-risk patients with coronary heart disease and diabetes.

The CHAT and CHAT-DM studies are further distinguished by their large sample sizes and culturally appropriate, theory-driven text messages. Most text message intervention studies that have focused on health behavior or diabetes management have had sample sizes ranging from 18-357 participants.[53] CHAT has a sample size of around 790 while the sample size of CHAT-DM is 480. It is noteworthy that CHAT is the first study using theory-based text messaging as a method of delivery for CHD patients' behavior change in China. Data from other text messaging intervention studies suggest that text messaging programs achieved better results when message content is theory-based,[53] however, few text messaging studies specified a theoretical rationale. Further, text messages were tailored to a specific

For peer review only - http://bmjopen.bmj.com/site/about/guidelines.xhtml

Chinese patient population. Studies have found that the majority of Chinese adults prefer learning by following directive rules and guidelines,[54] and practical counseling instructions were provided through text messages to help them better modify health behaviors. Apart from this, the text message language was designed to be plain and easy to memorize, consistent with Chinese cultural features such as aphorism, chengyu and catchy rhyme schemes, making it more acceptable to patients.

The two studies have several additional strengths. In contrast to most prior single center text-messaging trials, these two studies are conducted at 37 participating sites spread across a large and geographically diverse country. These results may be more generalizable. Moreover, our research team devoted significant attention to quality data collection, including rigorous on-site monitoring of questionnaire answers, samples collection, and data management. Furthermore, an evaluation of the acceptability and feasibility of our text message-based intervention will provide further important evidence to inform future studies, particularly with regards to optimizing text content, text frequency, and the overall user experience.

Our study also has some potential limitations. First, medication adherence and physical activity are measured by self-report, which carries the possibility of recall bias and social desirability bias. However, we believe that any such bias would be balanced across the treatment and control groups, and MMAS-8 and IPAQ have been validated and are widely used in measuring these metrics. Second, the text messages, though semi-personalized with participant's preferred name and depending on their smoking status, were not tailored specifically to every individual, which may reduce their efficacy. These

For peer review only - http://bmjopen.bmj.com/site/about/guidelines.xhtml

questions might be addressed in future studies using more individualized messages.

The CHAT and CHAT-DM studies have important public health implications. As low cost, non-pharmacological interventions, the CHAT and CHAT-DM studies may serve as important models for patient-centered, evidence-based public health interventions. LMICs, including China, face challenges with huge burdens of CVD and DM, limited health resources, and geographically and culturally diverse patient populations, making them the ideal places to conduct such studies. Still, high levels of mobile phone ownership across countries and income levels signal a promising new avenue for clinical research and that it may be possible to scale effective mobile health interventions for delivery to large populations in the coming years. Considering that text messages are low in cost and incur minimal, if any, risk, if this innovative and simple prevention were proven to be helpful, its benefits would be substantial in a country as large and populous as China.

In conclusion, the CHAT and CHAT-DM studies are multi-center, randomized controlled trials that are being conducted at 37 hospitals, and will thoroughly test the efficacy of text-messages to support secondary prevention for CHD and DM in China. The studies target high-risk patient populations with a culturally-sensitive, scalable and cost-effective text-message intervention. These two trials go through comprehensive data collection and rigorous data management, and have the potential to provide novel insights into disease management and be scaled-up to improve health in a significant proportion of patients in future.

For peer review only - http://bmjopen.bmj.com/site/about/guidelines.xhtml

Acknowledgments: We appreciate the multiple contributions made by study teams at the China Oxford Centre for International Health Research and the Yale-New Haven Hospital Center for Outcomes Research and Evaluation in the areas of study design and operations, particularly the contributions to the text message bank by Xuekun Wu, Si Xuan and Xiuling Wang, and data collection and analysis by Ying Sun, Chaoqun Wu, Xueke Bai, Jiamin Liu and Wuhanbilige Hundei. We appreciate the valuable advice from Clara Chow and her team in Sydney, Weigang Zhao, Geng Liu, Yuanlin Guo, Zhuo Xu and Zengwu Wang. We are also grateful for the support provided by the Chinese government.

Contributions: X.H, Q.D, P.H, X.Z, M.I, W.G, J.L, X.L, L.J: study concept design; X.H, E.S.S, Q.D, P.H, C.M, X.Z, H.Z, X.Y, J.A.S, F.A.M, H.M.K: drafting the initial manuscript and critical revision of the paper. All authors read and approved the final manuscript.

Funding: This project was supported by the Research Special Fund for Public Welfare Industry of Health (201502009) from the National Health and Family Planning Commission of China, the National Key Technology R&D Program (2015BAI12B01, 2015BAI12B02) from the Ministry of Science and Technology of China and the 111 Project (B16005). Dr Spatz is supported by grant K12HS023000 from the Agency for Healthcare Research and Quality Patient-Centered Outcomes Research Institute (PCORI) Mentored Career Development Program. The sponsors had no role in the preparation or approval of the manuscript.

For peer review only - http://bmjopen.bmj.com/site/about/guidelines.xhtml

Competing interests: Dr. Krumholz is a recipient of research agreements from Medtronic and from Johnson & Johnson (Janssen), through Yale University, to develop methods of clinical trial data sharing. He is also the recipient of a grant from the Food and Drug Administration and Medtronic to develop methods for post-market surveillance of medical devices; and is the founder of Hugo, a personal health information platform. The other authors have no potential conflicts to disclose.

Ethical approval: The central ethics committee at the China National Center for Cardiovascular Disease (NCCD) approved the CHAT and CHAT-DM studies. All collaborating hospitals accepted the central ethics approval except for 8, which obtained local approval by internal ethics committees.

REFERENCES

1. Gaede PH, Jepsen PV, Larsen JN, etc. The Steno-2 study. Intensive multifactorial intervention reduces the occurrence of cardiovascular disease in patients with type 2 diabetes. *Ugeskr Laeger*. 2003;165:2658-61.
2. Clark AM, Hartling L, Vandermeer B, etc. Secondary prevention programmes for coronary heart disease: a meta-regression showing the merits of shorter, generalist, primary care-based interventions. *Eur J Cardiovasc Prev Rehabil*. 2007;14:538-46.
3. Suaya JA, Shepard DS, Normand SL, etc. Use of cardiac rehabilitation by Medicare beneficiaries after myocardial infarction or coronary bypass surgery. *Circulation*. 2007;116:1653-62.
4. Jackevicius CA, Li P and Tu JV. Prevalence, Predictors, and Outcomes of Primary Nonadherence After Acute Myocardial Infarction. *Circulation*. 2008;117:1028-1036.

For peer review only - http://bmjopen.bmj.com/site/about/guidelines.xhtml

5. Akincigil A, Bowblis JR, Levin C, etc. Long-term adherence to evidence based secondary prevention therapies after acute myocardial infarction. *J Gen Intern Med*. 2008;23:115-21.

6. Jackevicius CA, Mamdani M and Tu JV. Adherence with statin therapy in elderly patients with and without acute coronary syndromes. *JAMA*. 2002;288:462-7.

7. Desai NR and Choudhry NK. Impediments to adherence to post myocardial infarction medications. *Curr Cardiol Rep*. 2013;15:322.

8. Yusuf S, Islam S, Chow CK, etc. Use of secondary prevention drugs for cardiovascular disease in the community in high-income, middle-income, and low-income countries (the PURE Study): a prospective epidemiological survey. *Lancet*. 2011;378:1231-43.

9. Krueger KP, Berger BA and Felkey B. Medication adherence and persistence: a comprehensive review. *Adv Ther*. 2005;22:313-56.

10. Urquhart J. The odds of the three nons when an aptly prescribed medicine isn't working: non-compliance, non-absorption, non-response. *Br J Clin Pharmacol*. 2002;54:212-20.

11. Walsh JM, Sundaram V, McDonald K, etc. Implementing effective hypertension quality improvement strategies: barriers and potential solutions. *J Clin Hypertens (Greenwich)*. 2008;10:311-6.

12. Peyrot M, Rubin RR, Lauritzen T, etc. Psychosocial problems and barriers to improved diabetes management: results of the Cross-National Diabetes Attitudes, Wishes and Needs (DAWN) Study. *Diabet Med*. 2005;22:1379-85.

13. Renders CM, Valk GD, Griffin S, etc. Interventions to improve the management of diabetes mellitus in primary care, outpatient and community settings. *Cochrane Database Syst Rev*. 2001:CD001481.

14. Portal TS. https://www.statista.com/statistics/274774/forecast-of-mobile-phone-users-worldwide/ (accessed 20 Octorber 2016).

15. Union. IT. The World in 2010: ICT Facts and Figures. https://www.itu.int/ITU-D/ ict/material/FactsFigures2010.pdf 2010. 2010 (accessed 16 November 2016).

16. Ministry of Industry and Information Techonology of the People's Republic of China. Telecommunication industry situation of ecnomic operation in August

For peer review only - http://bmjopen.bmj.com/site/about/guidelines.xhtml

2016.https://www.miit.gov.cn/n1146312/n1146904/n1648372/c5295254/content.html. (accessed 10 November 2016).

17. RA A and I G-U. The role of mobile phones in increasing accessibility and efficiency in healthcare. *Vodafone Group Plc*. 2006:12-17.

18. Gurol-Urganci I, de Jongh T, Vodopivec-Jamsek V, etc. Mobile phone messaging reminders for attendance at healthcare appointments. *Cochrane Database Syst Rev*. 2013:CD007458.

19. Franklin VL, Waller A, Pagliari C, etc. A randomized controlled trial of Sweet Talk, a text-messaging system to support young people with diabetes. *Diabet Med*. 2006;23:1332-8.

20. Mills KT, Rubinstein A, Irazola V, etc. Comprehensive approach for hypertension control in low-income populations: rationale and study design for the hypertension control program in Argentina. *Am J Med Sci*. 2014;348:139-45.

21. Park LG, Howie-Esquivel J, Chung ML, etc. A text messaging intervention to promote medication adherence for patients with coronary heart disease: a randomized controlled trial. *Patient education and counseling*. 2014;94:261-8.

22. Free C, Knight R, Robertson S, etc. Smoking cessation support delivered via mobile phone text messaging (txt2stop): a single-blind, randomised trial. *Lancet*. 2011;378:49-55.

23. Maddison R, Whittaker R, Stewart R, etc. HEART: heart exercise and remote technologies: a randomized controlled trial study protocol. *BMC cardiovascular disorders*. 2011;11:26.

24. Muller AM, Alley S, Schoeppe S, etc. The effectiveness of e-& mHealth interventions to promote physical activity and healthy diets in developing countries: A systematic review. *Int J Behav Nutr Phys Act.* 2016;13:109.

25. Chow CK, Redfern J, Hillis GS, etc. Effect of Lifestyle-Focused Text Messaging on Risk Factor Modification in Patients With Coronary Heart Disease: A Randomized Clinical Trial. *JAMA*. 2015;314:1255-63.

26. Arora S, Peters AL, Burner E, etc. Trial to examine text message-based mHealth in emergency department patients with diabetes (TExT-MED): a randomized controlled trial. *Ann Emerg Med*. 2014;63:745-54 e6.

27. Patrick K, Raab F, Adams MA, etc. A text message-based intervention for weight loss: randomized controlled trial. *J Med Internet Res*. 2009;11:e1.

For peer review only

28. Hurling R, Catt M, Boni MD, etc. Using internet and mobile phone technology to deliver an automated physical activity program: randomized controlled trial. *J Med Internet Res*. 2007;9:e7.

29. Hall CS, Fottrell E, Wilkinson S, etc. Assessing the impact of mHealth interventions in low- and middle-income countries--what has been shown to work? *Glob Health Action.* 2014;7:25606.

30. [Chinese guidelines for prevention of cardiovascular diseases]. *Zhonghua Xin Xue Guan Bing Za Zhi*. 2011;39:3-22.

31. Wang JG. Chinese Hypertension Guidelines. *Pulse*. 2015;3:14-20.

32. Weng J, Ji L, Jia W, etc. Standards of care for type 2 diabetes in China. *Diabetes/Metabolism Research and Reviews*. 2016;32:442-458.

33. Abraham C and Michie S. A taxonomy of behavior change techniques used in interventions. *Health psychology: official journal of the Division of Health Psychology, American Psychological Association*. 2008;27:379-87.

34. Michie S, Ashford S, Sniehotta FF, et al. A refined taxonomy of behaviour change techniques to help people change their physical activity and healthy eating behaviours: the CALO-RE taxonomy. *Psychol Health.* 2011;26:1479-98.

35. Michie S, Richardson M, Johnston M, et al. The behavior change technique taxonomy (v1) of 93 hierarchically clustered techniques: building an international consensus for the reporting of behavior change interventions. *Ann Behav Med.* 2013;46:81-95.

36. Gelin M, Bugaiska A, Meot A , etc. Are animacy effects in episodic memory independent of encoding instructions? *Memory*. 2017;25:2-18.

37. Lin YN. The application of cognitive-behavioral therapy to counseling Chinese. *American journal of psychotherapy*. 2002;56:46-58.

38. Exum HA and Lau EYw. Counseling style preference of Chinese college students. *Journal of Multicultural Counseling and Development*. 1988;16:84-92.

39. Yeung GT and Fung HH. Social support and life satisfaction among Hong Kong Chinese older adults: family first? *European Journal of Ageing*. 2007;4:219-227.

40. Tseng W-S and Wu DY. *Chinese culture and mental health*: Academic Press; 2013.

41. Morisky DE, Green LW and Levine DM. Concurrent and predictive validity

For peer review only - http://bmjopen.bmj.com/site/about/guidelines.xhtml

of a self-reported measure of medication adherence. *Med Care*. 1986;24:67-74.

42. Group I. International physical activity questionnaire. 2002.

43. NN Q and KJ L. Study on the reliability and validity of international physical activity questionnaire(Chinese vision,IPAQ). *Chin J Epidemiol*. 2004;25:265-268.

44. Chan PS, Jones PG, Arnold SA, etc. Development and validation of a short version of the Seattle angina questionnaire. *Circ Cardiovasc Qual Outcomes*. 2014;7:640-7.

45. Wang H, Kindig DA and Mullahy J. Variation in Chinese population health related quality of life: results from a EuroQol study in Beijing, China. *Qual Life Res*. 2005;14:119-32.

46. Stevens KR and Munoz LR. Cigarette smoking: Evidence to guide measurement. *Res Nurs Health*. 2004;27:281-92.

47. Parker DR, Lasater TM, Windsor R, etc. The accuracy of self-reported smoking status assessed by cotinine test strips. *Nicotine Tob Res*. 2002;4:305-9.

48. Hu D, Li J, Li X, etc. Investigation of blood lipid levels and statin interventions in outpatients with coronary heart disease in China: the China Cholesterol Education Program (CCEP). *Circ J*. 2008;72:2040-5.

49. Bi Y, Zhu D, Cheng J, etc. The status of glycemic control: A cross-sectional study of outpatients with type 2 diabetes mellitus across primary, secondary, and tertiary hospitals in the Jiangsu province of China. *Clin Ther*. 2010;32:973-83.

50. Jinping Z, Lixia J, Wenhui Z, etc. Relationship between personal medical care and microvascular complications in patients with type 2 diabetes mellitus in Beijing. *Chin J Diabetes Mellitus*. 2016;8:463-467.

51. Smith SC, Jr. Current and future directions of cardiovascular risk prediction. *Am J Cardiol*. 2006;97:28A-32A.

52. Weingarten SR, Henning JM, Badamgarav E, etc. Interventions used in disease management programmes for patients with chronic illness-which ones work? Meta-analysis of published reports. *BMJ*. 2002;325:925.

53. Hall AK, Cole-Lewis H and Bernhardt JM. Mobile Text Messaging for Health: A Systematic Review of Reviews. *Annual review of public health*.

For peer review only - http://bmjopen.bmj.com/site/about/guidelines.xhtml

2015;36:393-415.
54. Littrell R. Teaching students from Confucian cultures. 2005.

For peer review only
For peer review only - http://bmjopen.bmj.com/site/about/guidelines.xhtml

Figure Legends

Figure 1. Geographic distribution of sites in CHAT and CHAT-DM studies

Figure 2. Text Development Process

For peer review only

For peer review only - http://bmjopen.bmj.com/site/about/guidelines.xhtml

Table 1. Behavior change techniques used in message development and example messages

Behavior change technique[33]	Content/explanation[33]	Example text message in English
Provide information about behavior-health link	General information about behavioral risk	The way you cook can impact your health as well. Steaming, boiling and sautéing are better ways to cook than deep-frying and pan-frying. Cooking in less oil is a healthy alternative.
Prompt barrier identification	Identify barriers to perform the behavior and plan ways of overcoming them	Taking diabetes medications and injecting insulin regularly can help control your blood sugar. Forgetting to take your medication? Try to set a repeating alarm on your cell phone to remind you to take your medication or insulin injection.
Set graded tasks	Set easy tasks, and increase difficulty until target behavior is reached	Have you been finding it hard to quit smoking? In the beginning things are always hard. You can use a schedule to gradually reduce the number of cigarettes you smoke. For example, you

For peer review only - http://bmjopen.bmj.com/site/about/guidelines.xhtml

		may try going from 20 cigarettes to 15 per day for a week.
Provide instruction	Tell the person how to perform a behavior and/or preparatory behaviors	If you experience symptoms of angina (severe chest pain), place one nitroglycerin tablet under your tongue. Sit, stay calm and rest if you ever forget medications while going out. If angina symptoms are not relieved within 10 minutes, seek medical attention immediately.
Prompt self-monitoring of behavior	The person is asked to keep a record of specified behavior(s)	A cold or diarrhea will make your blood sugar levels rise, so monitor your glucose more frequently when you are sick. If you are using insulin, test your blood glucose 6-8 times a day, keep a blood glucose log and share it with your health providers.
Prompt practice	Prompt the person to rehearse and repeat the behavior or preparatory behaviors	As an old Chinese saying goes, 'It takes more than one cold day for a river to freeze three feet deep; ice in the river takes a long time to melt.' Similarly, cerebrovascular disease requires

For peer review only - http://bmjopen.bmj.com/site/about/guidelines.xhtml

		long-term prevention and treatment. Remember to take your medications as prescribed!
Plan social support or social change	Prompt consideration of how others could change their behavior to offer the person help or (instrumental) social support	Quitting smoking on your own can be difficult. Tell your friends and family when you are quitting so that they will stop giving you cigarettes. Support and encouragement from your loved one can be helpful as well.
Stress management	May involve a variety of specific techniques (e.g., progressive relaxation) that do not target the behavior but seek to reduce anxiety and stress	Relaxation is something we need to learn and practice. Listening to music, reading, or talking to friends and family can ease stress.
Motivational interviewing	Prompt the person to provide self-motivating statements and	Did you smoke less today than you did yesterday or days before? If you did reduce the amount of cigarettes, it is something worth

For peer review only

For peer review only - http://bmjopen.bmj.com/site/about/guidelines.xhtml

evaluations of their own behaviors to minimize resistance to change	celebrating. We are sure that you have put a lot of effort into quitting. Keep up the good work and you can make a difference!

For peer review only

For peer review only - http://bmjopen.bmj.com/site/about/guidelines.xhtml

Table 2. Survey scores for various message categories in user test

	Strongly agree or agree	Neutral	Strongly disagree or disagree
**General health messages**			
Information easy to understand (n=142)	130 (91.6%)	6 (4.2%)	6 (4.2%)
Information was useful (n=142)	129 (90.9%)	9 (6.3%)	4 (2.8%)
**Hypertension**			
Information easy to understand (n=181)	168 (92.8%)	7 (3.9%)	6 (3.3%)
Information was useful (n=181)	169 (93.4%)	6 (3.3%)	6 (3.3%)
**Medication Adherence**			
Information easy to understand (n=149)	134 (89.9%)	10 (6.7%)	5 (3.4%)
Information was useful (n=149)	137 (92.0%)	10 (6.7%)	2 (1.3%)
**Physical Activity**			
Information easy to understand (n=116)	109 (93.9%)	6 (5.2%)	1 (0.9%)
Information was useful	111 (95.7%)	4 (3.4%)	1 (0.9%)

For peer review only - http://bmjopen.bmj.com/site/about/guidelines.xhtml

(n=116)			
**Smoking cessation**			
Information easy to understand (n=42)	41 (97.6%)	1 (2.4%)	0 (0%)
Information was useful (n=42)	41 (97.6%)	0 (0%)	1 (2.4%)

For peer review only

For peer review only - http://bmjopen.bmj.com/site/about/guidelines.xhtml

Table 3. Example Text Messages for the CHAT and CHAT-DM Studies

CHAT TEXT [6 texts/wk]		CHAT-DM TEXT [6 texts/wk]
**General Education (CVD) [2x/wk]*** The most common risk factors for coronary artery disease are smoking, obesity, high blood pressure, high cholesterol and diabetes. However, most of them can be controlled in an appropriate way.		**General Education (DM) [1x/wk]** Diabetes is not terrible and there are many things you can do to prevent problems from diabetes, such as monitoring blood glucose, watching your diet, keeping fit, and taking pills regularly.
**Blood Pressure Control [2x/wk]** Most people do not experience any symptoms of high blood pressure. Do not stop taking blood pressure medication unless directed by your doctor. It is important for patients with hypertension to take medication diligently and to monitor their blood pressure on a regular basis.		**Blood Pressure Control [1x/wk]** Home Blood Pressure Monitoring is highly recommended! You can get an accurate picture of your heart health and understand daily changes in blood pressure, which is helpful for doctors to adjust medications for you.
**Medication Adherence [1x/wk]**		**Medication Adherence [1x/wk]**

For peer review only - http://bmjopen.bmj.com/site/about/guidelines.xhtml

For peer review only

Do you have a problem remembering to take your blood pressure medications? If so, try to tell your family about your medicine schedule so they can remind you.	Talk to doctors about your concerns and any uncomfortable symptoms after taking pills. Let your doctor help you to find the right medication for you.
**Physical Activity [1x/wk]**<br>You can still choose low-intensity exercise even after heart attack, such as walking and Tai Chi, at a slower pace and stick to your exercise plan. Always consult your physician before beginning any exercise program.	**Physical Activity [1x/wk]**<br>Try brisk walking – a convenient, safe and cost-effective way of exercising! It's good for your heart and will help control blood glucose.
**Smoking Cessation [1x/wk]**[†]<br>Do you worry about your family having health problems because of your smoking? Quitting is an important choice you can make to benefit your family's health, too. Secondhand smoke can cause respiratory disease, lung cancer, and heart disease.	**Diabetes Management [1x/wk]**<br>See a doctor before you travel. Always carry your diabetes medications and insulin, glucose meter, and strips, so that you can better monitor your glucose. Carry some hard candy and crackers to avoid low sugar.

For peer review only - http://bmjopen.bmj.com/site/about/guidelines.xhtml

		**Life style intervention [1x/wk]**<br>Individuals with diabetes should consume a balanced diet, and eat smaller but more frequent meals. Consider splitting your meal and save it for a snack later. Some healthy snack choices include tomatoes, cucumbers, and sugar-free biscuits.

*** In CHAT, non-smokers receive two General Education messages per week, while smokers only receive one per week.**

† In CHAT, only smokers receive smoking cessation messages.

For peer review only - http://bmjopen.bmj.com/site/about/guidelines.xhtml

Table 4. Baseline and follow-up data collection

Information	Baseline	6 month
Basic and contact information	√	√
Physical examination: BP, HR, waist circumference, weight, height	√	√
Ambulatory blood pressure	√	√
Outcome (death, myocardial infarction, angina, stroke, revascularization, etc.)	√	√
Hospitalizations	√	√
Current medications	√	√
Medication adherence (Morisky)	√	√
Physical activity (IPAQ)	√	√
CVD functional status (SAQ)	√	√
Health status (EQ-5D)	√	√
Socioeconomic status	√	√
Risk factors control	√	√
Urine cotinine/nicotine test	√	√
Blood and urine sample for core lab and local test	√	√

For peer review only - http://bmjopen.bmj.com/site/about/guidelines.xhtml

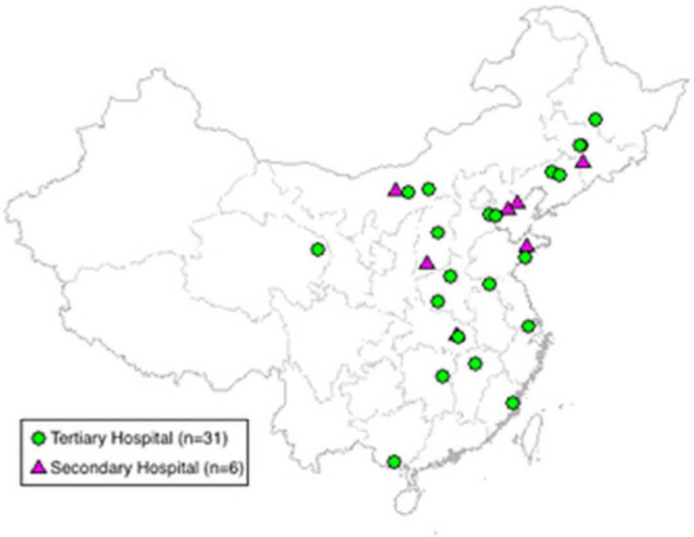

Figure 1. Geographic distribution of sites in CHAT and CHAT-DM studies

110x72mm (300 x 300 DPI)

review only

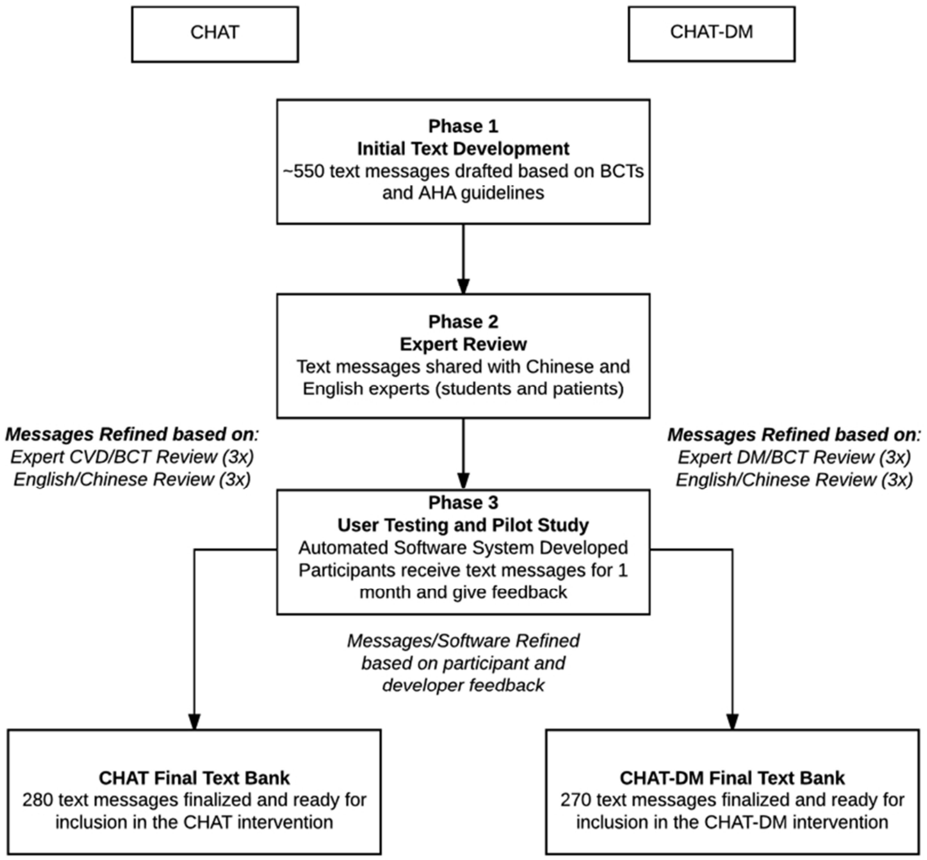

Figure 2. Text Development Process

For peer review only - http://bmjopen.bmj.com/site/about/guidelines.xhtml

Supplementary Materials

Design and rationale of the Cardiovascular Health and Text Messaging (CHAT) Study and CHAT-Diabetes Mellitus (CHAT-DM) Study: two randomized controlled trials of text messaging to improve secondary prevention for coronary heart disease and diabetes

TABLE OF CONTENTS

Page 2-3: CHAT and CHAT-DM studies site investigators by hospital

For peer review only

CHAT and CHAT-DM studies site investigators by hospital

1. China-Japan Union Hospital of Jilin University, Ping Yang, Zhaohui Feng, Mei Ding, Bing Li;
2. Dongfeng County Hospital, Wei Liu, Xiaoxin Li, Yanbin Zhang, Wen Cui, Sen Shi;
3. First Hospital of Shanxi Medical University, Qinghua Han, Liqin Duan, Chunrong Jin;
4. General Hospital of China FAW Group Corporation, Hongtao Pan, Lei Sun, Peng Chen;
5. Inner Mongolia Baogang Hopital, Yongdong Li, Liqing Jia, Xingxing Ren;
6. Jiangxi Provincial People's Hospital, Lang Hong, Ji Hong, Linfeng Li, Lihua Yuan, Yun Li;
7. Laixi People's Hospital, Xu Jiang, Hui Liu ,Guohao Xu, Yanrong Song;
8. Laoting County Hospital, Keyong Shang, Changjiang Liu, Kuituan Xi, Ying Yu;
9. Lujiang County People's Hospital, Kelian Xu, Cunqi Wang, Yongan Qian;
10. Nanyang Central Hospital, Shouzhong Yang, Yudong Li, Jianbu Gao, Songyu Zhang;
11. Port Hospital of Hebei Port Group Company, Ltd, Xia Wu, Penghui Yang, Xueqing Wang;
12. Qingdao Fuwai Hospital, Xianyan Jiang, Bin Zhang, Yumei Dong, Cheng Zheng, Wenchuan Hu;
13. Qinghai Cardiovascular and Cerebrovascular Hospital, Huiping Bian, Bo Chen, Xiaojuan Han, Na Han;
14. Qinzhou Second People's Hospital, Liyuan Chen, Qiuxia Liu, Lin Chen;
15. Quwo County People's Hospital, Xiwu Wang, Zhenlin Wu, Qiang Wang, Zhaohui Shangguan;
16. Shanxi Cardiovascular Hospital, Bin Yang, Yingting Yang;
17. Shenyang the Fourth Hospital of People, Yinjun Li, Wei Jiang;
18. Shuangcheng People's Hospital, Cheng Zhang, Aiguo Sun, Xuemei Wang;
19. Suzhou Kowloon Hospital, Shanghai Jiao Tong University School of Medicine, Feng Liu, Qiaoxia Zhou, Bingbing Zhu, Jie Guo;
20. The Affiliated Hospital of Qingdao University, Changyong Zhou, Yini Wang, Tao Yu, Zhe Su;
21. The First Affiliated Hospital of Fujian Medical University, Jinxiu Lin, Dajun Chai, Qinghui Guo;
22. The Affiliated Hospital of Xuzhou Medical University, Dongye Li, Yuanyuan Luo, Junhong Chen, Wei Qian, Qian Yu, Lulu Sun;
23. The First Hospital of Jilin University, Yang Zheng, Zhaoxi Liu, Lin Zou;
24. The Fourth Affiliated Hospital of China Medical University, Yuanzhe Jin, Xiaohong Zhang, Weina Hu;
25. The people's hospital of Dongxihu district of Wuhan ,Yongbo Lin, Ling Zhou, Hanliang Dan;
26. The People's Hospital of Liaoning Province, Zhanquan Li, Ying Liu, Dan Li;

For peer review only

27. The Second Affiliated Hospital of Xuzhou Medical College, Weiheng Wu, Li Li, Sai Zhang;
28. The Second Affiliated Hospital of Zhengzhou University, Xianen Fa, Lihua Zhang, Liqiang Sun, Youxu Jiang;
29. Tianjin Medical University General Hospital, Yuemin Sun, Bo Bian;
30. TEDA International Cardiovascular Hospital, Zhigang Liu, Zhipeng Guo, Cun Zhang;
31. Tongji Hospital of Tongji Medical College, Huazhong University of Science and Technology, Jiangang Jiang, Xiaoqing Shen, Ting Yu;
32. Wuhan Asia Heart Hospital, Xi Su, Songzhi Zhao, Wei Wu, Yujing Fan;
33. Wulate County People's Hospital, Jinlan Xu, Lei Xia, Yunmei Wang;
34. Xiangtan Central Hospital, He Huang, Jianping Zeng, Mingxing Wu, Yi Zhou;
35. Xinmin People's Hospital, Bo Jiang, Liwei Qi, Tongying Li;
36. Xuzhou First People's Hospital, Hongju Zhang, Chen Bian, Wei Li;
37. Zhengzhou Central Hospital, Lin Zhang, Yumei Guo;

For peer review only

SPIRIT

STANDARD PROTOCOL ITEMS: RECOMMENDATIONS FOR INTERVENTIONAL TRIALS

SPIRIT 2013 Checklist: Recommended items to address in a clinical trial protocol and related documents*

Section/item	Item No	Description	Addressed on page number
**Administrative information**			
Title	1	Descriptive title identifying the study design, population, interventions, and, if applicable, trial acronym	1
Trial registration	2a	Trial identifier and registry name. If not yet registered, name of intended registry	3
	2b	All items from the World Health Organization Trial Registration Data Set	N/A
Protocol version	3	Date and version identifier	N/A
Funding	4	Sources and types of financial, material, and other support	23
Roles and responsibilities	5a	Names, affiliations, and roles of protocol contributors	1-2
	5b	Name and contact information for the trial sponsor	23
	5c	Role of study sponsor and funders, if any, in study design; collection, management, analysis, and interpretation of data; writing of the report; and the decision to submit the report for publication, including whether they will have ultimate authority over any of these activities	23
	5d	Composition, roles, and responsibilities of the coordinating centre, steering committee, endpoint adjudication committee, data management team, and other individuals or groups overseeing the trial, if applicable (see Item 21a for data monitoring committee)	23-24

For peer review only - http://bmjopen.bmj.com/site/about/guidelines.xhtml

For peer review only

Introduction

Background and rationale	6a	Description of research question and justification for undertaking the trial, including summary of relevant studies (published and unpublished) examining benefits and harms for each intervention	_____5-6_____
	6b	Explanation for choice of comparators	_____5-6_____
Objectives	7	Specific objectives or hypotheses	______7______
Trial design	8	Description of trial design including type of trial (eg, parallel group, crossover, factorial, single group), allocation ratio, and framework (eg, superiority, equivalence, noninferiority, exploratory)	______8-9____

Methods: Participants, interventions, and outcomes

Study setting	9	Description of study settings (eg, community clinic, academic hospital) and list of countries where data will be collected. Reference to where list of study sites can be obtained	______7______
Eligibility criteria	10	Inclusion and exclusion criteria for participants. If applicable, eligibility criteria for study centres and individuals who will perform the interventions (eg, surgeons, psychotherapists)	______8______
Interventions	11a	Interventions for each group with sufficient detail to allow replication, including how and when they will be administered	_____8-12____
	11b	Criteria for discontinuing or modifying allocated interventions for a given trial participant (eg, drug dose change in response to harms, participant request, or improving/worsening disease)	______9______
	11c	Strategies to improve adherence to intervention protocols, and any procedures for monitoring adherence (eg, drug tablet return, laboratory tests)	_____14______
	11d	Relevant concomitant care and interventions that are permitted or prohibited during the trial	______9______
Outcomes	12	Primary, secondary, and other outcomes, including the specific measurement variable (eg, systolic blood pressure), analysis metric (eg, change from baseline, final value, time to event), method of aggregation (eg, median, proportion), and time point for each outcome. Explanation of the clinical relevance of chosen efficacy and harm outcomes is strongly recommended	____16-17____
Participant timeline	13	Time schedule of enrolment, interventions (including any run-ins and washouts), assessments, and visits for participants. A schematic diagram is highly recommended (see Figure)	______7______

For peer review only - http://bmjopen.bmj.com/site/about/guidelines.xhtml

Sample size	14	Estimated number of participants needed to achieve study objectives and how it was determined, including clinical and statistical assumptions supporting any sample size calculations	____18-19____
Recruitment	15	Strategies for achieving adequate participant enrolment to reach target sample size	______8______
**Methods: Assignment of interventions (for controlled trials)**			
Allocation:			
Sequence generation	16a	Method of generating the allocation sequence (eg, computer-generated random numbers), and list of any factors for stratification. To reduce predictability of a random sequence, details of any planned restriction (eg, blocking) should be provided in a separate document that is unavailable to those who enrol participants or assign interventions	_____8-9_____
Allocation concealment mechanism	16b	Mechanism of implementing the allocation sequence (eg, central telephone; sequentially numbered, opaque, sealed envelopes), describing any steps to conceal the sequence until interventions are assigned	______9______
Implementation	16c	Who will generate the allocation sequence, who will enrol participants, and who will assign participants to interventions	_____8-9_____
Blinding (masking)	17a	Who will be blinded after assignment to interventions (eg, trial participants, care providers, outcome assessors, data analysts), and how	______9______
	17b	If blinded, circumstances under which unblinding is permissible, and procedure for revealing a participant's allocated intervention during the trial	____ N/A _____
**Methods: Data collection, management, and analysis**			
Data collection methods	18a	Plans for assessment and collection of outcome, baseline, and other trial data, including any related processes to promote data quality (eg, duplicate measurements, training of assessors) and a description of study instruments (eg, questionnaires, laboratory tests) along with their reliability and validity, if known. Reference to where data collection forms can be found, if not in the protocol	____14-15____
	18b	Plans to promote participant retention and complete follow-up, including list of any outcome data to be collected for participants who discontinue or deviate from intervention protocols	____ N/A _____

For peer review only - http://bmjopen.bmj.com/site/about/guidelines.xhtml

Data management	19	Plans for data entry, coding, security, and storage, including any related processes to promote data quality (eg, double data entry; range checks for data values). Reference to where details of data management procedures can be found, if not in the protocol	____15-16____
Statistical methods	20a	Statistical methods for analysing primary and secondary outcomes. Reference to where other details of the statistical analysis plan can be found, if not in the protocol	____18-19____
	20b	Methods for any additional analyses (eg, subgroup and adjusted analyses)	_____18______
	20c	Definition of analysis population relating to protocol non-adherence (eg, as randomised analysis), and any statistical methods to handle missing data (eg, multiple imputation)	____ N/A _____
**Methods: Monitoring**			
Data monitoring	21a	Composition of data monitoring committee (DMC); summary of its role and reporting structure; statement of whether it is independent from the sponsor and competing interests; and reference to where further details about its charter can be found, if not in the protocol. Alternatively, an explanation of why a DMC is not needed	____ N/A _____
	21b	Description of any interim analyses and stopping guidelines, including who will have access to these interim results and make the final decision to terminate the trial	____ N/A _____
Harms	22	Plans for collecting, assessing, reporting, and managing solicited and spontaneously reported adverse events and other unintended effects of trial interventions or trial conduct	_____15______
Auditing	23	Frequency and procedures for auditing trial conduct, if any, and whether the process will be independent from investigators and the sponsor	____ N/A _____
**Ethics and dissemination**			
Research ethics approval	24	Plans for seeking research ethics committee/institutional review board (REC/IRB) approval	_____24______
Protocol amendments	25	Plans for communicating important protocol modifications (eg, changes to eligibility criteria, outcomes, analyses) to relevant parties (eg, investigators, REC/IRBs, trial participants, trial registries, journals, regulators)	____ N/A _____

For peer review only - http://bmjopen.bmj.com/site/about/guidelines.xhtml

Consent or assent	26a	Who will obtain informed consent or assent from potential trial participants or authorised surrogates, and how (see Item 32)	______7______
	26b	Additional consent provisions for collection and use of participant data and biological specimens in ancillary studies, if applicable	____ N/A _____
Confidentiality	27	How personal information about potential and enrolled participants will be collected, shared, and maintained in order to protect confidentiality before, during, and after the trial	____15-16____
Declaration of interests	28	Financial and other competing interests for principal investigators for the overall trial and each study site	_____24______
Access to data	29	Statement of who will have access to the final trial dataset, and disclosure of contractual agreements that limit such access for investigators	____15-16____
Ancillary and post-trial care	30	Provisions, if any, for ancillary and post-trial care, and for compensation to those who suffer harm from trial participation	____ N/A _____
Dissemination policy	31a	Plans for investigators and sponsor to communicate trial results to participants, healthcare professionals, the public, and other relevant groups (eg, via publication, reporting in results databases, or other data sharing arrangements), including any publication restrictions	_____19______
	31b	Authorship eligibility guidelines and any intended use of professional writers	_____23______
	31c	Plans, if any, for granting public access to the full protocol, participant-level dataset, and statistical code	____ N/A _____
**Appendices**			
Informed consent materials	32	Model consent form and other related documentation given to participants and authorised surrogates	____ N/A _____
Biological specimens	33	Plans for collection, laboratory evaluation, and storage of biological specimens for genetic or molecular analysis in the current trial and for future use in ancillary studies, if applicable	_____N/A _____

*It is strongly recommended that this checklist be read in conjunction with the SPIRIT 2013 Explanation & Elaboration for important clarification on the items. Amendments to the protocol should be tracked and dated. The SPIRIT checklist is copyrighted by the SPIRIT Group under the Creative Commons "Attribution-NonCommercial-NoDerivs 3.0 Unported" license.

For peer review only - http://bmjopen.bmj.com/site/about/guidelines.xhtml

Design and rationale of the Cardiovascular Health and Text Messaging (CHAT) Study and CHAT-Diabetes Mellitus (CHAT-DM) Study: two randomized controlled trials of text messaging to improve secondary prevention for coronary heart disease and diabetes

Journal:	*BMJ Open*
Manuscript ID	bmjopen-2017-018302.R2
Article Type:	Protocol
Date Submitted by the Author:	24-Oct-2017
Complete List of Authors:	Huo, Xiqian; Fuwai Hospital, National Center for Cardiovascular Diseases, State Key Laboratory of Cardiovascular Disease<br>Spatz, Erica; Yale University<br>Ding, Qinglan; Yale University<br>Horak, Paul ; Yale University<br>Zheng, Xin; Fuwai Hospital, National Center for Cardiovascular Diseases, State Key Laboratory of Cardiovascular Disease<br>Masters, Claire ; Yale University<br>Zhang, Haibo; Cardiovascular Institute & Fu Wai Hospital, Chinese Academy of Medical Sciences & Peking Union Medical College, China Oxford Centre for International Health Research<br>Irwin, Melinda; Yale School of Public Health<br>Yan, Xiaofang; Fuwai Hospital, National Center for Cardiovascular Diseases, State Key Laboratory of Cardiovascular Disease<br>Guan, Wenchi; Cardiovascular Institute & Fu Wai Hospital, Chinese Academy of Medical Sciences & Peking Union Medical College, China Oxford Centre for International Health Research,<br>Li, Jing; Cardiovascular Institute & Fu Wai Hospital, Chinese Academy of Medical Sciences & Peking Union Medical College, China Oxford Centre for International Health Research<br>Li, Xi; Fuwai Hospital, National Center for Cardiovascular Diseases, State Key Laboratory of Cardiovascular Disease<br>Spertus, John; Mid America Heart Institute of Saint Luke's Hospital<br>Masoudi, Frederick; Division of Cardiology, University of Colorado Anschutz Medical Campus<br>Krumholz, Harlan; Yale School of Public Health<br>Jiang, Lixin; Fuwai Hospital, National Center for Cardiovascular Diseases, State Key Laboratory of Cardiovascular Disease
**Primary Subject Heading**:	Research methods
Secondary Subject Heading:	Research methods, Medical management
Keywords:	Coronary heart disease < CARDIOLOGY, diabetes, text messaging, behavioral intervention, secondary prevention

Note: The following files were submitted by the author for peer review, but cannot be converted to PDF. You must view these files (e.g. movies) online.

bmjopen-2017-018302_Revised manuscript_updated.docx

SCHOLARONE™ Manuscripts

For peer review only

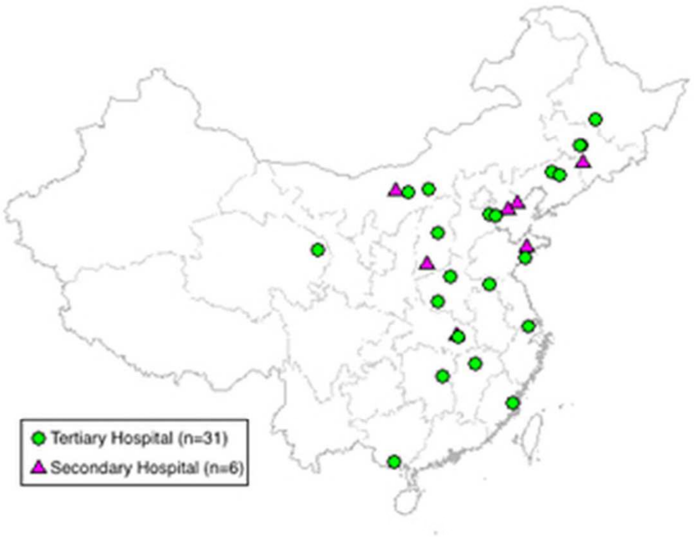

Figure 1. Geographic distribution of sites in CHAT and CHAT-DM studies

110x72mm (300 x 300 DPI)

For peer review only

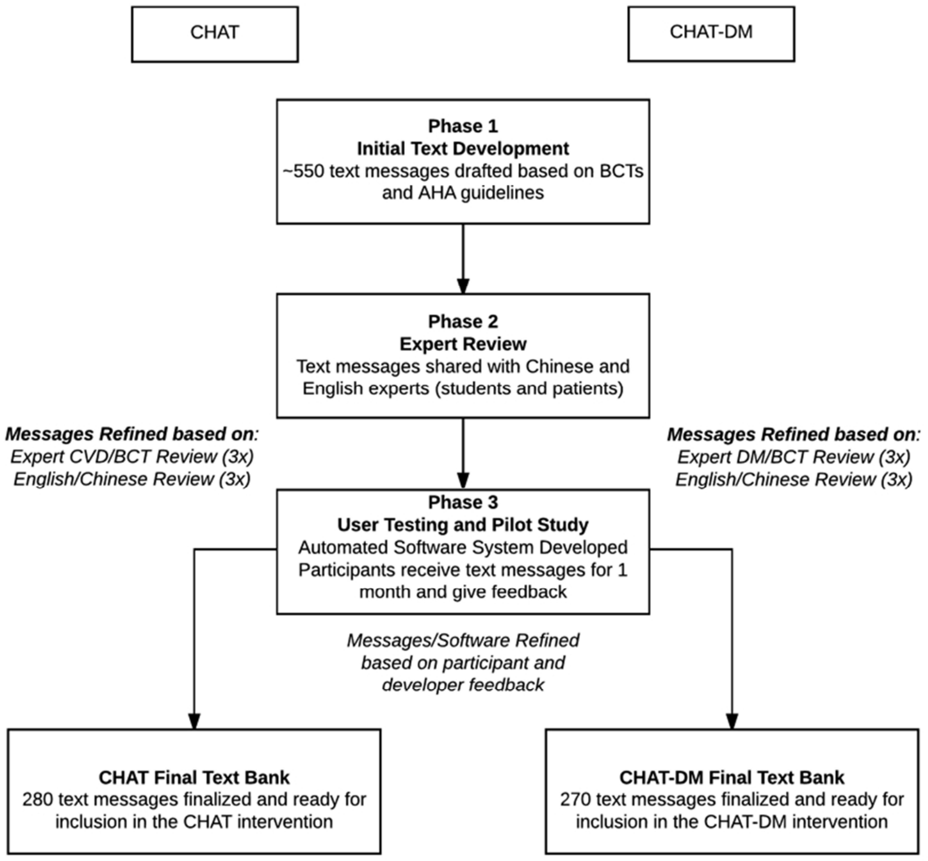

Figure 2. Text Development Process

For peer review only

Supplementary Materials

Design and rationale of the Cardiovascular Health and Text Messaging (CHAT) Study and CHAT-Diabetes Mellitus (CHAT-DM) Study: two randomized controlled trials of text messaging to improve secondary prevention for coronary heart disease and diabetes

TABLE OF CONTENTS

Page 2-3: CHAT and CHAT-DM studies site investigators by hospital

For peer review only

CHAT and CHAT-DM studies site investigators by hospital

1. China-Japan Union Hospital of Jilin University, Ping Yang, Zhaohui Feng, Mei Ding, Bing Li;
2. Dongfeng County Hospital, Wei Liu, Xiaoxin Li, Yanbin Zhang, Wen Cui, Sen Shi;
3. First Hospital of Shanxi Medical University, Qinghua Han, Liqin Duan, Chunrong Jin;
4. General Hospital of China FAW Group Corporation, Hongtao Pan, Lei Sun, Peng Chen;
5. Inner Mongolia Baogang Hopital, Yongdong Li, Liqing Jia, Xingxing Ren;
6. Jiangxi Provincial People's Hospital, Lang Hong, Ji Hong, Linfeng Li, Lihua Yuan, Yun Li;
7. Laixi People's Hospital, Xu Jiang, Hui Liu ,Guohao Xu, Yanrong Song;
8. Laoting County Hospital, Keyong Shang, Changjiang Liu, Kuituan Xi, Ying Yu;
9. Lujiang County People's Hospital, Kelian Xu, Cunqi Wang, Yongan Qian;
10. Nanyang Central Hospital, Shouzhong Yang, Yudong Li, Jianbu Gao, Songyu Zhang;
11. Port Hospital of Hebei Port Group Company, Ltd, Xia Wu, Penghui Yang, Xueqing Wang;
12. Qingdao Fuwai Hospital, Xianyan Jiang, Bin Zhang, Yumei Dong, Cheng Zheng, Wenchuan Hu;
13. Qinghai Cardiovascular and Cerebrovascular Hospital, Huiping Bian, Bo Chen, Xiaojuan Han, Na Han;
14. Qinzhou Second People's Hospital, Liyuan Chen, Qiuxia Liu, Lin Chen;
15. Quwo County People's Hospital, Xiwu Wang, Zhenlin Wu, Qiang Wang, Zhaohui Shangguan;
16. Shanxi Cardiovascular Hospital, Bin Yang, Yingting Yang;
17. Shenyang the Fourth Hospital of People, Yinjun Li, Wei Jiang;
18. Shuangcheng People's Hospital, Cheng Zhang, Aiguo Sun, Xuemei Wang;
19. Suzhou Kowloon Hospital, Shanghai Jiao Tong University School of Medicine, Feng Liu, Qiaoxia Zhou, Bingbing Zhu, Jie Guo;
20. The Affiliated Hospital of Qingdao University, Changyong Zhou, Yini Wang, Tao Yu, Zhe Su;
21. The First Affiliated Hospital of Fujian Medical University, Jinxiu Lin, Dajun Chai, Qinghui Guo;
22. The Affiliated Hospital of Xuzhou Medical University, Dongye Li, Yuanyuan Luo, Junhong Chen, Wei Qian, Qian Yu, Lulu Sun;
23. The First Hospital of Jilin University, Yang Zheng, Zhaoxi Liu, Lin Zou;
24. The Fourth Affiliated Hospital of China Medical University, Yuanzhe Jin, Xiaohong Zhang, Weina Hu;
25. The people's hospital of Dongxihu district of Wuhan ,Yongbo Lin, Ling Zhou, Hanliang Dan;
26. The People's Hospital of Liaoning Province, Zhanquan Li, Ying Liu, Dan Li;

For peer review only

27. The Second Affiliated Hospital of Xuzhou Medical College, Weiheng Wu, Li Li, Sai Zhang;
28. The Second Affiliated Hospital of Zhengzhou University, Xianen Fa, Lihua Zhang, Liqiang Sun, Youxu Jiang;
29. Tianjin Medical University General Hospital, Yuemin Sun, Bo Bian;
30. TEDA International Cardiovascular Hospital, Zhigang Liu, Zhipeng Guo, Cun Zhang;
31. Tongji Hospital of Tongji Medical College, Huazhong University of Science and Technology, Jiangang Jiang, Xiaoqing Shen, Ting Yu;
32. Wuhan Asia Heart Hospital, Xi Su, Songzhi Zhao, Wei Wu, Yujing Fan;
33. Wulate County People's Hospital, Jinlan Xu, Lei Xia, Yunmei Wang;
34. Xiangtan Central Hospital, He Huang, Jianping Zeng, Mingxing Wu, Yi Zhou;
35. Xinmin People's Hospital, Bo Jiang, Liwei Qi, Tongying Li;
36. Xuzhou First People's Hospital, Hongju Zhang, Chen Bian, Wei Li;
37. Zhengzhou Central Hospital, Lin Zhang, Yumei Guo;

For peer review only

SPIRIT 2013 Checklist: Recommended items to address in a clinical trial protocol and related documents*

Section/item	Item No	Description	Addressed on page number
**Administrative information**			
Title	1	Descriptive title identifying the study design, population, interventions, and, if applicable, trial acronym	______1_______
Trial registration	2a	Trial identifier and registry name. If not yet registered, name of intended registry	______3_______
	2b	All items from the World Health Organization Trial Registration Data Set	____ N/A _____
Protocol version	3	Date and version identifier	____ N/A _____
Funding	4	Sources and types of financial, material, and other support	______23______
Roles and responsibilities	5a	Names, affiliations, and roles of protocol contributors	____ 1-2______
	5b	Name and contact information for the trial sponsor	______23______
	5c	Role of study sponsor and funders, if any, in study design; collection, management, analysis, and interpretation of data; writing of the report; and the decision to submit the report for publication, including whether they will have ultimate authority over any of these activities	______23 _____
	5d	Composition, roles, and responsibilities of the coordinating centre, steering committee, endpoint adjudication committee, data management team, and other individuals or groups overseeing the trial, if applicable (see Item 21a for data monitoring committee)	____23-24____

For peer review only - http://bmjopen.bmj.com/site/about/guidelines.xhtml

**Introduction**			
Background and rationale	6a	Description of research question and justification for undertaking the trial, including summary of relevant studies (published and unpublished) examining benefits and harms for each intervention	_____5-6_____
	6b	Explanation for choice of comparators	_____5-6_____
Objectives	7	Specific objectives or hypotheses	______7______
Trial design	8	Description of trial design including type of trial (eg, parallel group, crossover, factorial, single group), allocation ratio, and framework (eg, superiority, equivalence, noninferiority, exploratory)	______8-9____
**Methods: Participants, interventions, and outcomes**			
Study setting	9	Description of study settings (eg, community clinic, academic hospital) and list of countries where data will be collected. Reference to where list of study sites can be obtained	______7______
Eligibility criteria	10	Inclusion and exclusion criteria for participants. If applicable, eligibility criteria for study centres and individuals who will perform the interventions (eg, surgeons, psychotherapists)	______8______
Interventions	11a	Interventions for each group with sufficient detail to allow replication, including how and when they will be administered	_____8-12____
	11b	Criteria for discontinuing or modifying allocated interventions for a given trial participant (eg, drug dose change in response to harms, participant request, or improving/worsening disease)	______9______
	11c	Strategies to improve adherence to intervention protocols, and any procedures for monitoring adherence (eg, drug tablet return, laboratory tests)	_____14______
	11d	Relevant concomitant care and interventions that are permitted or prohibited during the trial	______9______
Outcomes	12	Primary, secondary, and other outcomes, including the specific measurement variable (eg, systolic blood pressure), analysis metric (eg, change from baseline, final value, time to event), method of aggregation (eg, median, proportion), and time point for each outcome. Explanation of the clinical relevance of chosen efficacy and harm outcomes is strongly recommended	____16-17____
Participant timeline	13	Time schedule of enrolment, interventions (including any run-ins and washouts), assessments, and visits for participants. A schematic diagram is highly recommended (see Figure)	______7______

For peer review only - http://bmjopen.bmj.com/site/about/guidelines.xhtml

Sample size	14	Estimated number of participants needed to achieve study objectives and how it was determined, including clinical and statistical assumptions supporting any sample size calculations	____18-19____
Recruitment	15	Strategies for achieving adequate participant enrolment to reach target sample size	______8______

Methods: Assignment of interventions (for controlled trials)

Allocation:			
Sequence generation	16a	Method of generating the allocation sequence (eg, computer-generated random numbers), and list of any factors for stratification. To reduce predictability of a random sequence, details of any planned restriction (eg, blocking) should be provided in a separate document that is unavailable to those who enrol participants or assign interventions	_____8-9_____
Allocation concealment mechanism	16b	Mechanism of implementing the allocation sequence (eg, central telephone; sequentially numbered, opaque, sealed envelopes), describing any steps to conceal the sequence until interventions are assigned	______9______
Implementation	16c	Who will generate the allocation sequence, who will enrol participants, and who will assign participants to interventions	_____8-9_____
Blinding (masking)	17a	Who will be blinded after assignment to interventions (eg, trial participants, care providers, outcome assessors, data analysts), and how	______9______
	17b	If blinded, circumstances under which unblinding is permissible, and procedure for revealing a participant's allocated intervention during the trial	____ N/A _____

Methods: Data collection, management, and analysis

Data collection methods	18a	Plans for assessment and collection of outcome, baseline, and other trial data, including any related processes to promote data quality (eg, duplicate measurements, training of assessors) and a description of study instruments (eg, questionnaires, laboratory tests) along with their reliability and validity, if known. Reference to where data collection forms can be found, if not in the protocol	____14-15____
	18b	Plans to promote participant retention and complete follow-up, including list of any outcome data to be collected for participants who discontinue or deviate from intervention protocols	____ N/A _____

For peer review only - http://bmjopen.bmj.com/site/about/guidelines.xhtml

Data management	19	Plans for data entry, coding, security, and storage, including any related processes to promote data quality (eg, double data entry; range checks for data values). Reference to where details of data management procedures can be found, if not in the protocol	____15-16____
Statistical methods	20a	Statistical methods for analysing primary and secondary outcomes. Reference to where other details of the statistical analysis plan can be found, if not in the protocol	____18-19____
	20b	Methods for any additional analyses (eg, subgroup and adjusted analyses)	_____18______
	20c	Definition of analysis population relating to protocol non-adherence (eg, as randomised analysis), and any statistical methods to handle missing data (eg, multiple imputation)	____ N/A _____
**Methods: Monitoring**			
Data monitoring	21a	Composition of data monitoring committee (DMC); summary of its role and reporting structure; statement of whether it is independent from the sponsor and competing interests; and reference to where further details about its charter can be found, if not in the protocol. Alternatively, an explanation of why a DMC is not needed	____ N/A _____
	21b	Description of any interim analyses and stopping guidelines, including who will have access to these interim results and make the final decision to terminate the trial	____ N/A _____
Harms	22	Plans for collecting, assessing, reporting, and managing solicited and spontaneously reported adverse events and other unintended effects of trial interventions or trial conduct	_____15______
Auditing	23	Frequency and procedures for auditing trial conduct, if any, and whether the process will be independent from investigators and the sponsor	____ N/A _____
**Ethics and dissemination**			
Research ethics approval	24	Plans for seeking research ethics committee/institutional review board (REC/IRB) approval	_____24______
Protocol amendments	25	Plans for communicating important protocol modifications (eg, changes to eligibility criteria, outcomes, analyses) to relevant parties (eg, investigators, REC/IRBs, trial participants, trial registries, journals, regulators)	____ N/A _____

For peer review only - http://bmjopen.bmj.com/site/about/guidelines.xhtml

Consent or assent	26a	Who will obtain informed consent or assent from potential trial participants or authorised surrogates, and how (see Item 32)	______7______
	26b	Additional consent provisions for collection and use of participant data and biological specimens in ancillary studies, if applicable	____ N/A _____
Confidentiality	27	How personal information about potential and enrolled participants will be collected, shared, and maintained in order to protect confidentiality before, during, and after the trial	____15-16____
Declaration of interests	28	Financial and other competing interests for principal investigators for the overall trial and each study site	_____24______
Access to data	29	Statement of who will have access to the final trial dataset, and disclosure of contractual agreements that limit such access for investigators	____15-16____
Ancillary and post-trial care	30	Provisions, if any, for ancillary and post-trial care, and for compensation to those who suffer harm from trial participation	____ N/A _____
Dissemination policy	31a	Plans for investigators and sponsor to communicate trial results to participants, healthcare professionals, the public, and other relevant groups (eg, via publication, reporting in results databases, or other data sharing arrangements), including any publication restrictions	_____19______
	31b	Authorship eligibility guidelines and any intended use of professional writers	_____23______
	31c	Plans, if any, for granting public access to the full protocol, participant-level dataset, and statistical code	____ N/A _____
**Appendices**			
Informed consent materials	32	Model consent form and other related documentation given to participants and authorised surrogates	Supplementary. 1
Biological specimens	33	Plans for collection, laboratory evaluation, and storage of biological specimens for genetic or molecular analysis in the current trial and for future use in ancillary studies, if applicable	_____N/A _____

*It is strongly recommended that this checklist be read in conjunction with the SPIRIT 2013 Explanation & Elaboration for important clarification on the items. Amendments to the protocol should be tracked and dated. The SPIRIT checklist is copyrighted by the SPIRIT Group under the Creative Commons "Attribution-NonCommercial-NoDerivs 3.0 Unported" license.

For review only

For peer review only - http://bmjopen.bmj.com/site/about/guidelines.xhtml
